# Two New Species of *Lophozia* (Marchantiophyta) from the Sino-Himalaya and the Taxonomic Diversity of East Asian *Lophozia*

**DOI:** 10.3390/plants14192997

**Published:** 2025-09-27

**Authors:** Vadim A. Bakalin, Yulia D. Maltseva, Ksenia G. Klimova, Wenzhang Ma, Seung Se Choi

**Affiliations:** 1 Laboratory of Cryptogamic Biota, Botanical Garden-Institute of the Far Eastern Branch of the Russian Academy of Sciences, 690024 Vladivostok, Russia; maltseva.yu.dm@gmail.com (Y.D.M.); ksenia.g.klimova@mail.ru (K.G.K.); 2 Herbarium, Key Laboratory for Plant Diversity and Biogeography of East Asia, Kunming Institute of Botany, Chinese Academy of Sciences, Kunming 650201, China; mawenzhang@mail.kib.ac.cn; 3 Team of National Ecosystem Survey, National Institute of Ecology, Seocheon 33657, Republic of Korea

**Keywords:** *Lophozia*, Sino-Himalaya, East Asia, Lophoziaceae, ecology, plagiotropic growth form, evolution, integrative taxonomy

## Abstract

An integrative study of material from Yunnan Province, China, revealed two new *Lophozia* species. These species and several other representatives of the genus known from East Asia form a distinct clade within the phylogenetic structure of *Lophozia*. Descriptions, photographs, and comments regarding the morphological characteristics of the new taxa are provided. *Lophozia neglecta* is characterized by pink gemmae (another taxon with similar gemmae is East Asian *L. koreana*), whereas *L. vinacea* is characterized by vine-purple gemmae, which were previously unknown in the genus. Additionally, molecular analysis confirmed the occurrence of *L. fuscovirens,* a poorly known *Lophozia* taxon with brown gemmae, in the Kamchatka Peninsula. The taxonomic diversity of *Lophozia* in East Asia comprises 12 species belonging to various distribution groups, including the Sino-Himalayan and broadly East Asian groups.

## 1. Introduction

Lophozia (Dumort.) Dumort. is not a species-rich genus of liverworts belonging to the order of the same name (Lophoziales). It is characterized by predominantly plagiotropic growth; relatively numerous oil bodies in leaf cells; bilobed (except for aberrations) leaves; tubular perianths that are conically narrowed to the tip, with a toothed mouth; and the presence of a microcellous layer in the stem, that is colonized by fungal hyphae. A distinct feature of *Lophozia* is its coexistence with fungi from Basidiomycota, characteristic of more evolutionarily advanced lines of leafy liverworts [1]. The latest compendium of *Lophozia* worldwide was provided by Söderström et al. [2]; it includes 16 species. Subsequently, taking into account the latest achievements, six more species were added to the mentioned revision. However, since some of the taxa have a dubious status, the exact number cannot be calculated at that moment, and currently, and it can be estimated at a preliminary level of 20–22 species [3]. Admittedly, the genus *Lophozia* is one of the most difficult in terms of morphological delimitation of species in the northern Holarctic [4,5]. However, it should be noted that the aforementioned difficulties [4,6] are observed precisely in the majority of northern Holarctic species, which are very abundant in northern latitudes, especially in the Hemiarctic and the corresponding altitudinal belt in the mountains north of 50° N. As vividly formulated by Schuster [4] (p. 549): “The “Ventricosae” [roughly equal to current *Lophozia* s. str.—VAB] are a large and incredibly difficult complex in which species concepts have not hardened. It is possible that, associated with sporadic gametophytic mutation and rather frequent sexual reproduction, the mechanisms exist in this group for rapid adaptive evolution, and the various “small species”—to use an old-fashioned term once current in hepaticology—and minor taxa may be relatively recent in origin”. However, whereas Schuster’s citation is fully applicable to the ‘northern’ taxa, the morphological differentiation of species found south of the North Holarctic, such as *L. dubia* Schiffn., *L. lacerata* N. Kitag., *L. lantratoviae* Bakalin, etc., is not very difficult [3]. At the same time, data on the species composition of East Asian *Lophozia* still appear incomplete. The latter may make discussions ambiguous. The Sino-Himalaya, especially its mountainous areas near and above the timberline, is a concentration of taxonomic liverwort diversity and may be a potentially promising region for *Lophozia* diversity as well. Such a ‘bloom’ of morphological and taxonomic diversity is known in the Sino-Himalaya, for example, in *Scapania* (Dumort.) Dumort. [7,8,9,10,11] and *Gymnomitrion* Corda [12,13,14]. Notably, both of the latter genera are quite diverse in the Hemiarctic as well [15]. There is no reason to assume that a similar phenomenon will not be observed in the genus *Lophozia*, which has thus far been very poorly studied in the Sino-Himalaya. Recent field studies in Yunnan Province, China, revealed unique plants resembling *Lophozia* and *Tritomaria* Schiffn. ex Loeske and inspired the work presented here, the main goal of which is a comprehensive description of the collected plants and an assessment of their phylogenetic position. In light of the new data obtained, it seems advisable to discuss how many species are known in East Asia and how these species are distributed. It is worth discussing whether they are limited to East Asia or parts of it, or are found more widely. In addition, the question arises whether some of the species found in East Asia belong to a separate line of evolution within the genus *Lophozia*. We also consider the presentation of such information to be one of the tasks of the presented account, although we acknowledge that the available data are still incomplete, which may influence the discussion results.

## 2. Results

### 2.1. Molecular Phylogenetic Reconstruction

Thirty-seven new accessions, six ITS1-2, seven *trn*L-*trn*F, six *trn*G, nine *rbc*L, and nine *rps*4, were produced and deposited into GenBank for the *Lophozia* specimens (Table A1). The lengths of the final alignments were 1367 (ITS1–2 + *trn*L–F), 1253 (*trn*G + *rps*4), and 1842 (*rbc*L + *rps*4), with 42 species sampled and the number of sequences 65, 44, and 34, respectively. The general characteristics of the alignments obtained during the phylogenetic analysis are shown in Table 1. The log-likelihood values for the ML analysis (Maximum Likelihood) and Bayesian analysis of all datasets are presented in Table 2.

**Figure 1:**
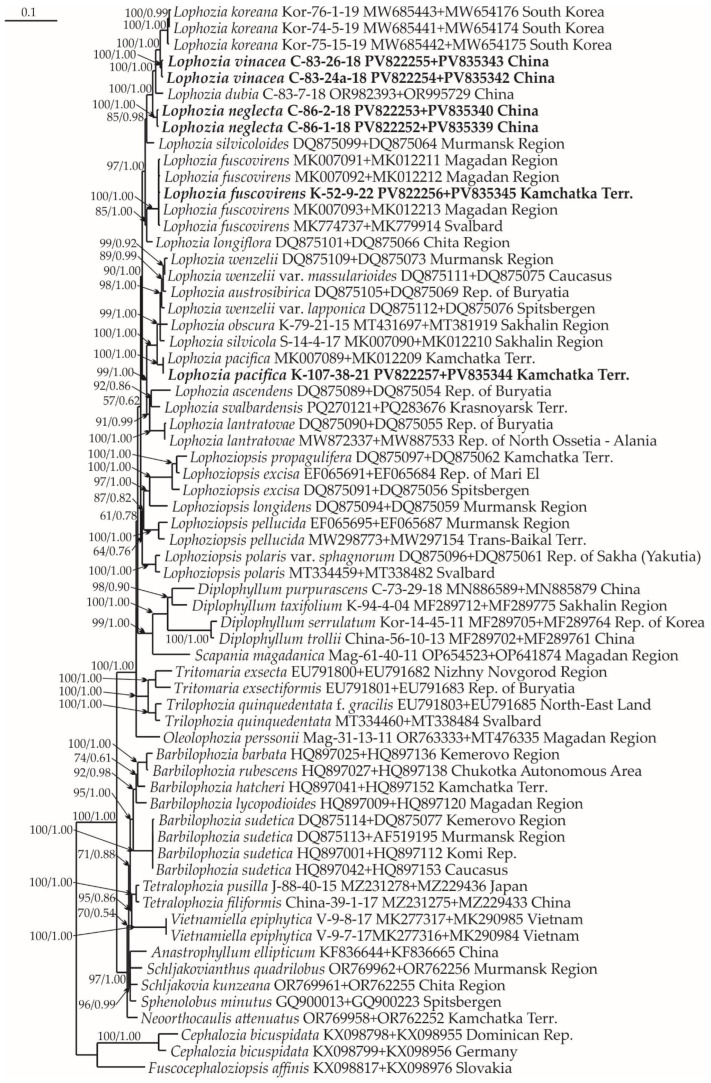
Phylogram obtained from a Bayesian analysis of the Lophoziaceae species and related taxa based on ITS 1-2—trnL-F. Newly obtained sequences are marked in bold. Bootstrap support values > 50% in ML analysis and Bayesian posterior probabilities PP > 0.50 are indicated. Scale bar denotes the number of nucleotide substitutions per site.

**Figure 2:**
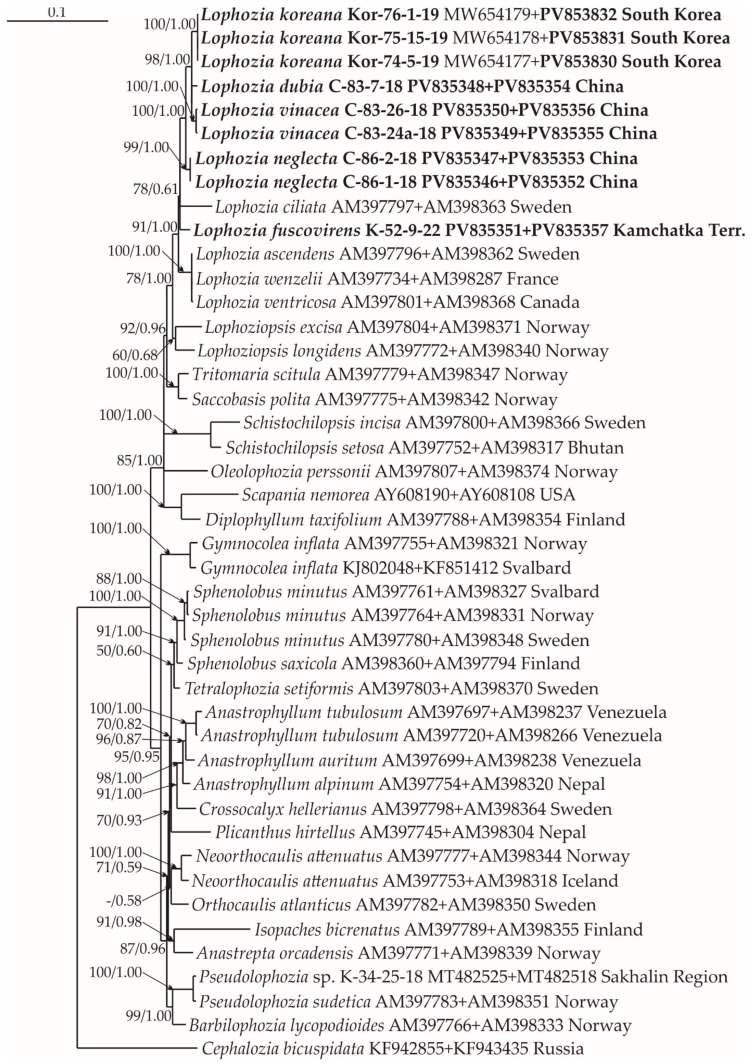
Phylogram obtained from a Bayesian analysis of the Lophoziaceae species and related taxa based on trnG-intron—rps4. Newly obtained sequences are marked in bold. Bootstrap support values > 50% in ML analysis and Bayesian posterior probabilities PP > 0.50 are indicated. Scale bar denotes the number of nucleotide substitutions per site.

**Figure 3:**
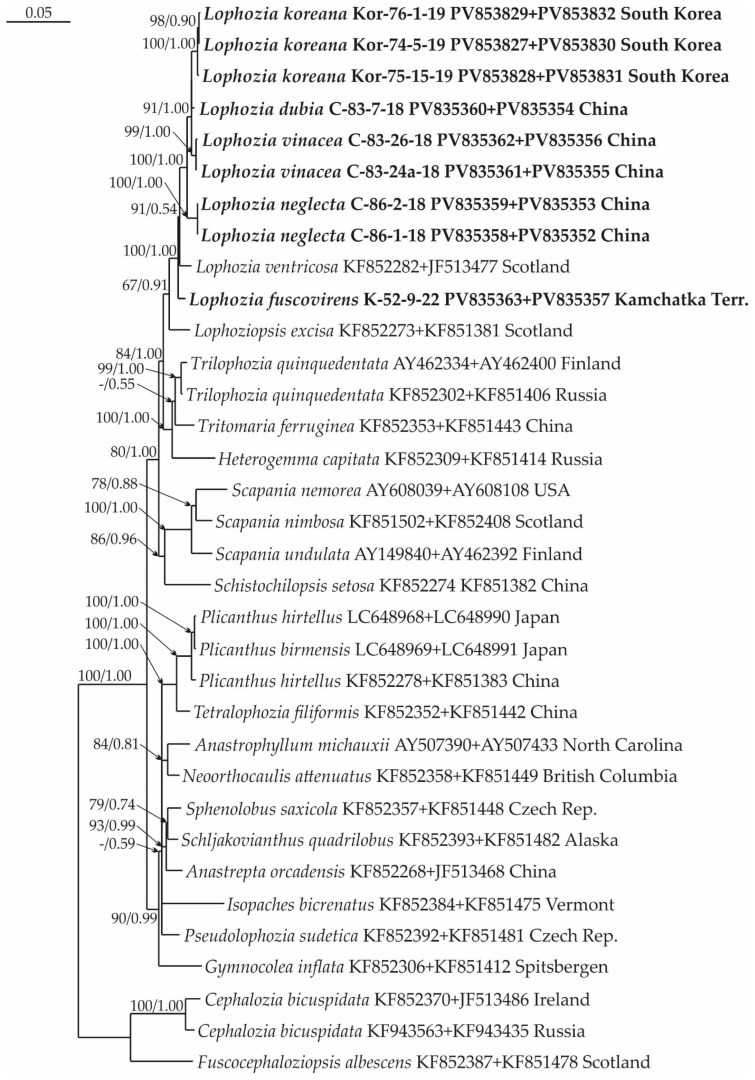
Phylogram obtained from a Bayesian analysis of the Lophoziaceae species and related taxa based on rbcL—rps4. Newly obtained sequences are marked in bold. Bootstrap support values > 50% in ML analysis and Bayesian posterior probabilities PP > 0.50 are indicated. Scale bar denotes the number of nucleotide substitutions per site.

The phylogenetic analysis revealed that some of the tested samples represent new species. These clades include the specimens C-83-24a-18 and C-83-26-18, treated here as *Lophozia vinacea*, and C-86-2-18 and C-86-1-18, treated here as *L. neglecta*. Both of these species are in isolated subclades on all trees (Figure 1, Figure 2 and Figure 3) and form a clade together with *L. koreana* (Bakalin, S.S. Choi et B.Y. Sun) Maltseva, Vilnet et Bakalin, and *L. dubia* inside the *Lophozia* clade. The divergence of *L. vinacea* and *L. neglecta* from other molecularly related species is provided in Table 3. It reached 2.79–5.67% for the ITS1–ITS2 locus, 0.25–3.2% for *trn*L–*trn*F, 0.87–2.6% for *trn*G, 0.38–1.61% for the *rbc*L, and 0.69–1.81% for the *rps*4 loci. This divergence level is similar to the difference between *Lophozia* species in neighboring clades (Table 3).

In addition to the two newly revealed taxa, the results of the analysis confirmed the occurrence of *L. fuscovirens* Bakalin et Vilnet in the Kamchatka Peninsula of Russia. Infraspecific *p*-distances within the *L. fuscovirens* clade in ITS1–ITS2 are 0.41 (Table 3); the specimen (K-52-9-22) belongs to the clade with other *L. fuscovirens* vouchers in Figure 1. This species was previously identified in Magadan Province, Russia, and Spitzbergen, Norway [16].

### 2.2. Taxonomy

*Lophozia neglecta* Bakalin, Maltseva, Klimova, S.S. Choi, W.Z. Ma sp. nov.

Description. Plants ascending to semierect in loose patches, in pure mats or intermixed with *Anastrepta orcadensis* (Hook.) Schiffn. and *Lophozia lantratovae*, brownish, yellowish brownish, with a purple tinged leaf base in its ventral part and a purple-brown ventral side of the stem, 10–20 mm long and 1.5–2.1 mm wide, sparsely intercalary branched. Stem cross section transversely ellipsoidal, in well-developed shoots ca. 320 × 400 μm, with large area (1/2–2/3 of stem height) occupied by the purple-colored small-celled zone colonized by fungal hyphae; external wall mostly thick, rarely thin, other walls thin, cells 20–25 μm in diameter across the stem cross section, with mostly small trigones, except in the microcellous layer, where only 5–10(–15) μm in diameter and free of trigones. Rhizoids abundant, obliquely to erect spreading, tightly attaching plants to the substratum and neighboring bryophytes, colorless to grayish, sometimes tinged with purple, especially in the distal parts of rhizoids. Leaves contiguous, obliquely to semierect spreading, subtransversely oriented, subtransversely to almost transversely inserted, canaliculate, sheathing the stem near base, when flattened on a slide ovate, 1.0–1.2 × 0.8–1.1 mm, divided by V- to, rarely, U-shaped sinus descending to 1/5–1/4(–1/3) of the leaf length, lobes acute, subequal, lobe apices erect spreading. Midleaf cells subisodiametric to shortly oblong, (20–25–30(–40) × (20–)25–30 μm, thin-walled, moderate in size, slightly convex to slightly concave, cuticle indistinctly striolate, oil bodies coarsely granulate, filling cell lumen; cells along margin 20–25 μm, thin-walled, but external wall thick, trigones mostly large, convex to slightly concave, cuticle smooth. Gemmae abundant, in clusters on leaf lobe apices of the 2–5(–8) upper pairs of leaves, colorless to pinkish (brownish in lower pairs of leaves when infested by fungi), (1–)2-celled, clearly angular with prominently thickened and protruding angles, in the projection 5–7-gonal, 12–15 × 10–13 μm, as long as wide or shortly oblong. Dioicous. Androecia intercalary, with 3–4 pairs of bracts, bracts ventricose in lower half and obliquely spreading above. Gynoecia (only unfertilized ones were seen) with 2–3 subfloral innovations, female bracts similar to leaves, bracteole not seen. Sporophytes and mature perianths unknown.

**Figure 4:**
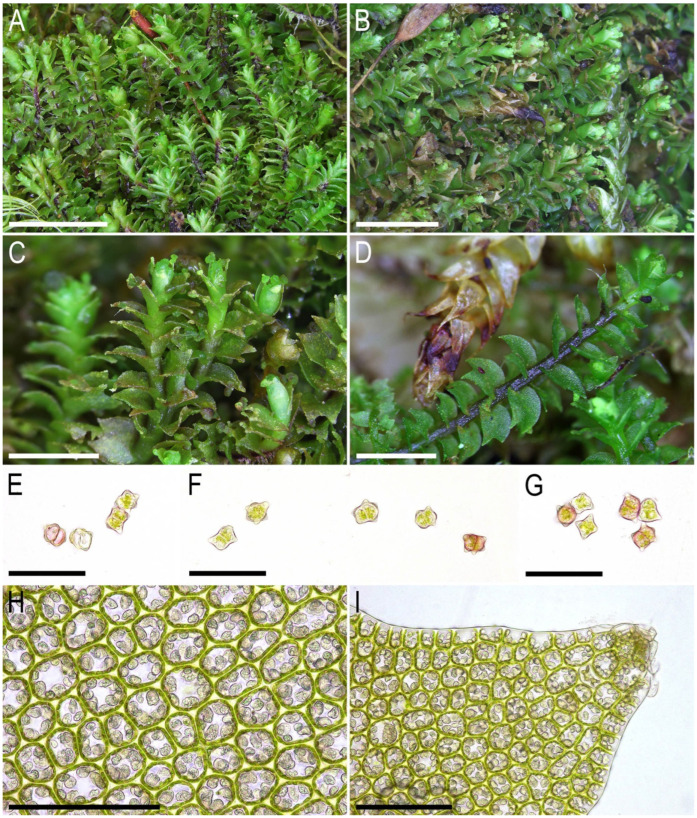
*Lophozia neglecta* Bakalin, Maltseva, Klimova, S.S. Choi, W.Z. Ma sp. nov. (all photographs were taken from plants in living conditions): (**A**,**B**) part of mat; (**C**) shoot with gemmae clusters, dorsal view; (**D**) shoot, dorsal view; (**E**–**G**) gemmae; (**H**) midleaf cells with oil bodies; (**I**) cells of leaf lobe with oil bodies. Scales: 5 mm for (**A**); 3 mm for (**B**); 2 mm for (**C**,**D**); 3 mm for (**D**); 50 µm for (**E**–**G**); 100 µm for (**H**,**I**). (**A**,**C**,**E**–**G**) from C-86-2-18 (VBGI), (**B**,**D**,**H**,**I**) from C-86-1-18 (VBGI).

**Figure 5:**
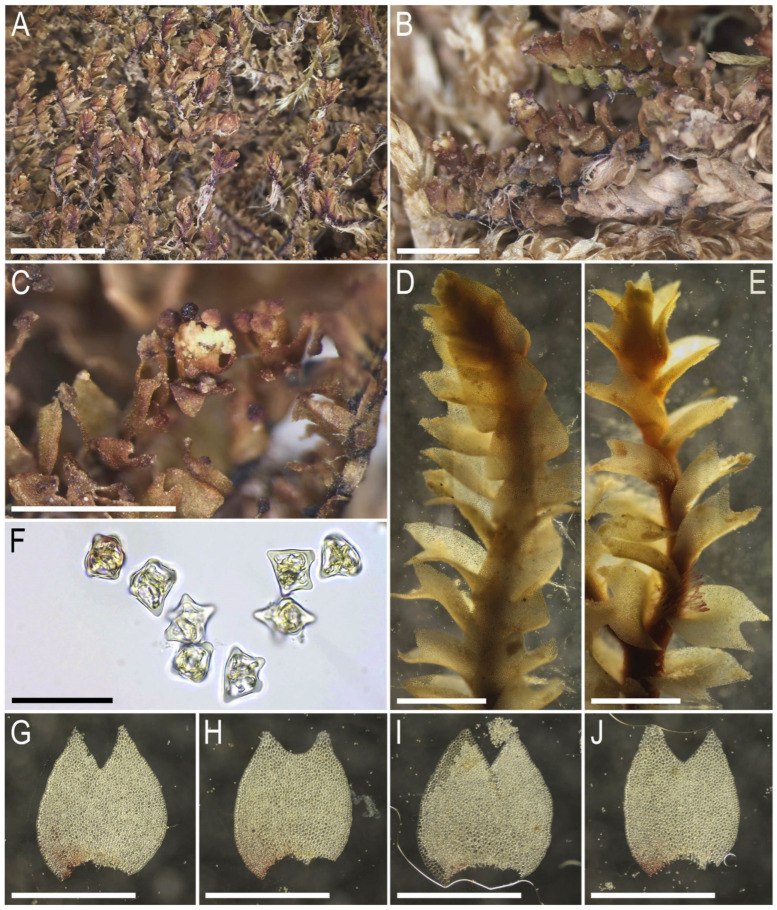
*Lophozia neglecta* Bakalin, Maltseva, Klimova, S.S. Choi, W.Z. Ma sp. nov. (all photographs were taken from herbarium material): (**A**) part of mat of dried plants; (**B**) dried shoots with gemmae clusters, lateral view; (**C**) dried shoot with gemmae clusters, dorsal view; (**D**) shoot, lateral view; (**E**) shoot, ventral view; (**F**) gemmae; (**G**–**J**) leaves; (**I**) leaf with gemmae cluster. Scales: 5 mm for (**A**); 3 mm for (**B**); 2 mm for (**C**,**D**); 3 mm for (**D**); 50 µm for (**E**–**G**); 100 µm for (**H**,**I**). All from C-86-2-18 (VBGI).

**Figure 6:**
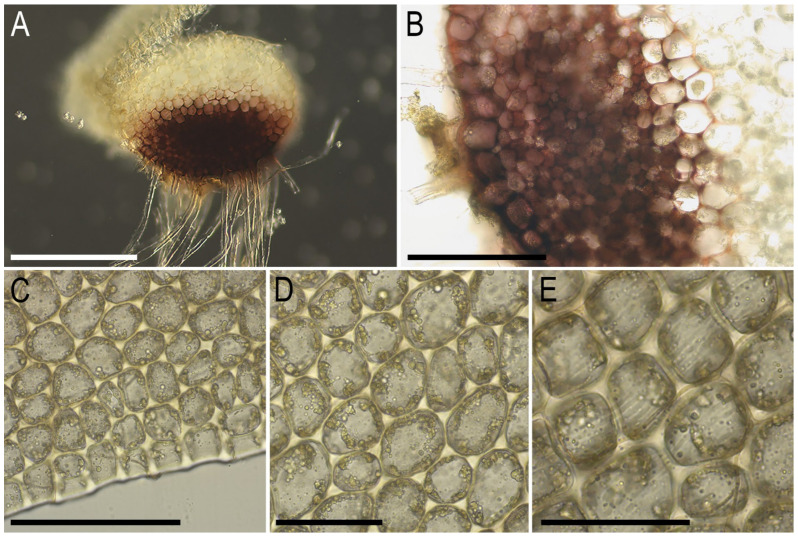
*Lophozia neglecta* Bakalin, Maltseva, Klimova, S.S. Choi, W.Z. Ma sp. nov. (all photographs were taken from herbarium material): (**A**) stem cross-section; (**B**) part of stem cross-section; (**C**) leaf margin cells with oil bodies; (**D**) midleaf cells with oil bodies; (**E**) unclearly striolate cuticle (leaf surface) in midleaf cells area. Scales: 300 µm for (**A**); 100 µm for (**B**,**C**); 50 µm for (**D**,**E**). All from C-86-2-18 (VBGI).

Illustrations in present paper: Figure 4, Figure 5 and Figure 6.

Holotype: CHINA. Yunnan Province, Diqing Tibetan Autonomous Prefecture, Shangri-La County, Xiao-Zhong-Dian Xiang, Bi-Gu Alpine Lakes area (27°37′26.4″ N 99°39′18.7″ E), 3847 m alt., coniferous (mostly *Picea* A. Dietr.) forest in the valley without permanent streams, moist partly shaded decaying wood, 17 October 2018, V.A. Bakalin & W.Z. Ma, C-86-2-18 (VBGI, isotype in KUN).

Paratype (virtually, part of the holotype): CHINA. Yunnan Province, Diqing Tibetan Autonomous Prefecture, Shangri-La County, Xiao-Zhong-Dian Xiang, Bi-Gu Alpine Lakes area (27°37′26.4″ N 99°39′18.7″ E), 3847 m alt., coniferous (mostly *Picea*) forest in the valley without permanent streams, moist partly shaded decaying wood, 17 October 2018, V.A. Bakalin & W.Z. Ma, C-86-1-18 (VBGI).

Comments. The most morphologically similar taxon, especially owing to the ability of gemmae to acquire pinkish pigmentation, is *Lophozia koreana* (originally described as *Tritomaria koreana* Bakalin, S.S. Choi et B.Y. Sun, position clarified by Bakalin et al. [17]). However, *L. neglecta* differs from the latter in the following ways:

1. Leaves have almost equal lobes (versus, as a rule, clearly unequal);
2. Leaf cells exhibit large trigones (versus small ones in *L. koreana*);
3. A well-defined microcellous layer on the cross section of the stem is present (Figure 6B) (in *L. koreana,* this layer is distinguishable only owing to color, but the size of the cells is almost the same as that in other parts of the stem cross section).
4. Plants are larger, exceeding 1.5 mm (versus 1.1–1.4 mm in width in *L. koreana*);
5. Coarsely papillose oil bodies fill the cell lumen (versus finely papillose oil bodies that do not fill the lumen of the cell in *L. koreana*);
6. The habitat is decaying wood (versus mineral soil in deep crevices for *L. koreana*);
7. Moreover, the new species has presumable Sino–Himalayan distribution (versus the mountainous Korean–Japanese distribution in *L. koreana*).

*Lophozia vinacea* Bakalin, Maltseva, Klimova, S.S. Choi, W.Z. Ma sp. nov.

Description. Plants ascending to erect, in rather loose patches, rarely in pure mats or intermixed with *Metacalypogeia alternifolia* (Nees) Grolle, *Syzygiella autumnalis* (DC.) K. Feldberg, Váňa, Hentschel et Heinrichs, *Blepharostoma* sp., *Schistochilopsis incisa* (Schrad.) Konstant., and other common epixylous taxa, brownish to rusty brown, ventral side of stem purple-black, ventral part of leaf base purplish, almost invariably with vinaceous purple clusters of gemmae within uppermost leaves, lobe apices turned toward the shoot apex, more or less rigid, branching not seen, 5–15 mm long and 1.5–2.0 mm wide. Stem brownish, on the ventral side deep purple, stem cross section slightly transversely ellipsoidal, with 2/3 of its height occupied by microcellous layer densely colonized by fungal hyphae, in well-developed shoots 250 × 290 μm, external wall thick, outer cells (12–)15–20 μm in diameter to oblong along section margin, 15–22 × 12–15 μm, with mostly thickened walls and large, concave trigones, inward become thinner-walled, with small, concave trigones, 15–22(–25) μm in diameter, in microcellous layer only 6–10 μm in diameter, thin-walled, with vestigial to absent trigones. Rhizoids dense, erect spreading, grayish brownish, tightly attaching the plant to the substratum or to neighboring bryophytes. Leaves contiguous to subimbricate, mostly subtransversely inserted and subtransversely to obliquely oriented, loosely sheathing the stem at the base and obliquely spreading above, concave-canaliculate, when flattened on a slide mainly ellipsoidal, rarely suborbicular and ovate, 1.0–1.2 × 0.8–1.1 mm, divided by widely V- to U-shaped sinus descending to 1/5(–1/4) of leaf length into two mostly unequal acute lobes. Midleaf cells subisodiametric to shortly oblong, 15–20(–30) × 15–20(–25) μm, thin-walled, trigones moderate in size, slightly convex, cuticle smooth to very indistinctly striolate; cells along margin 10–15 μm, walls thin, but external wall thickened, trigones moderate to large in size, mostly concave, cuticle smooth; cells in the leaf base elongate, 25–35 μm long, with more or less distinctly striolate cuticle to cuticle smooth. Gemmae vinaceous purple, among the uppermost leaves (not in clusters on the leaf lobe apices), small, 1(–2)-celled, 5–6-gonal, with prominently thickened angles. Generative structures unknown.

**Figure 7:**
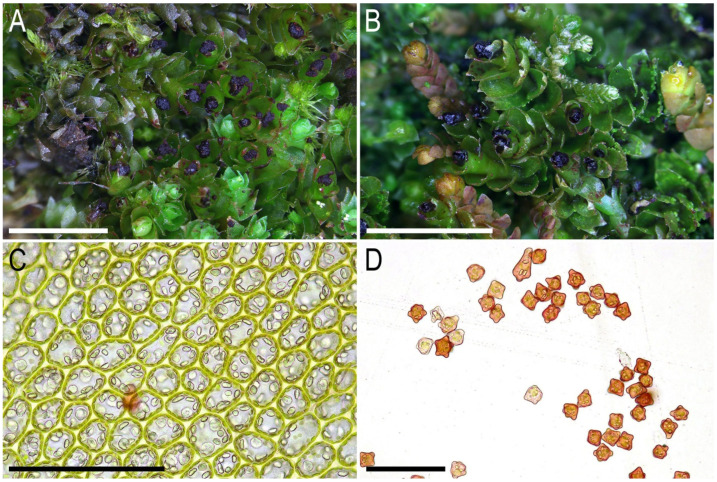
*Lophozia vinacea* Bakalin, Maltseva, Klimova, S.S. Choi, W.Z. Ma sp. nov. (all photographs were taken from plants in living conditions): (**A**) part of mat; (**B**) shoots with gemmae clusters; (**C**) midleaf cells with oil bodies; (**D**) gemmae. Scales: 3 mm for (**A**,**B**); 100 µm for (**C**); 50 µm for (**D**). (**A**,**C**,**D**) from C-83-24a-18 (VBGI), (**B**) from C-83-26-18 (VBGI).

**Figure 8:**
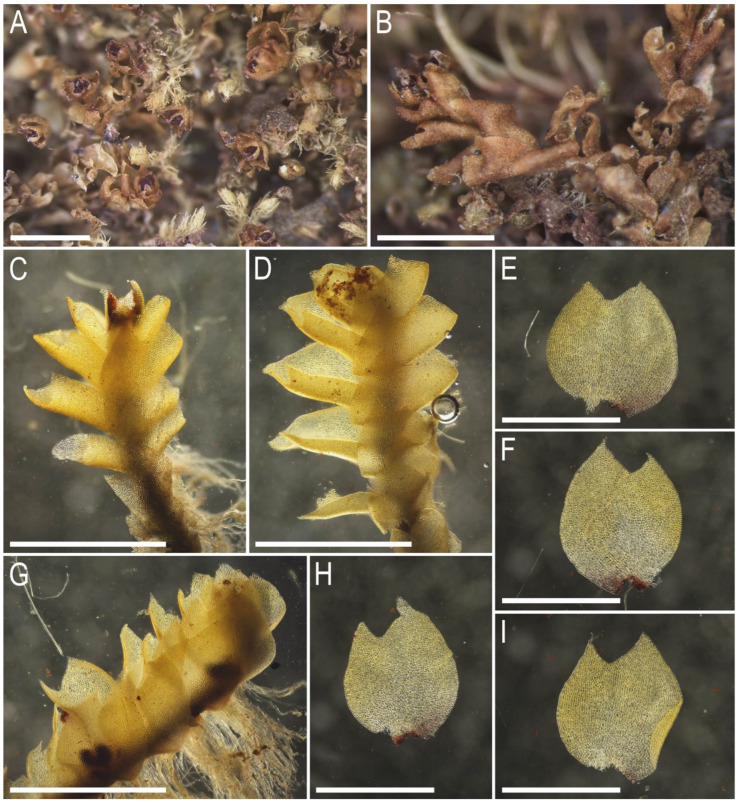
*Lophozia vinacea* Bakalin, Maltseva, Klimova, S.S. Choi, W.Z. Ma sp. nov. (all photographs were taken from herbarium material): (**A**) part of mat of dried plants; (**B**) dried shoot, dorsal view; (**C**,**D**) shoot with gemmae, lateral view; (**E**,**F**,**H**,**I**) leaves; (**G**) shoot with rhizoids, lateral view. Scales: 2 mm for (**A**); 1 mm for (**B**–**I**). All from C-83-26-18 (VBGI).

**Figure 9:**
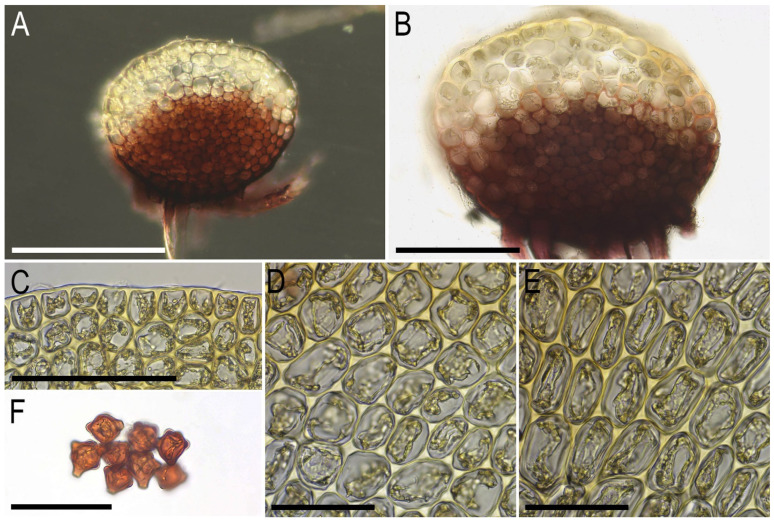
*Lophozia vinacea* Bakalin, Maltseva, Klimova, S.S. Choi, W.Z. Ma sp. nov. (all photographs were taken from herbarium material): (**A**,**B**) stem cross-section; (**C**) leaf margin cells; (**D**) midleaf cells; (**E**) elongate cells in the leaf base; (**F**) gemmae. Scales: 200 µm for (**A**); 100 µm for (**B**,**C**); 50 µm for (**D**–**F**). All from C-83-26-18 (VBGI).

Illustrations in present paper: Figure 7, Figure 8 and Figure 9.

Holotype: CHINA. Yunnan Province, Diqing Tibetan Autonomous Prefecture, Shangri-La County, Xiao-Zhong-Dian Xiang, Tian-Bao Mountain (27°36′55.6″ N 99°53′54.0″ E), 4031 m alt., narrow valley with coniferous forest with admixture of *Rhododendron* L. and many limestone outcrops (also resulting in a basic reaction of humificated soil), partly shaded moist decaying wood, 16 October 2018, V.A. Bakalin & W.Z. Ma, C-83-26-18 (VBGI, isotype in KUN).

Paratype (virtually, part of the holotype): CHINA. Yunnan Province, Diqing Tibetan Autonomous Prefecture, Shangri-La County, Xiao-Zhong-Dian Xiang, Tian-Bao Mountain (27°36′55.6″ N 99°53′54.0″ E), 4031 m alt., narrow valley with coniferous forest with admixture of *Rhododendron* and many limestone outcrops (also resulting in a basic reaction of humificated soil), partly shaded moist decaying wood, 16 October 2018, V.A. Bakalin & W.Z. Ma, C-83-24a-18 (VBGI).

Comments. Although the systematic position of the new species is definitely in *Lophozia* (Figure 1 and Figure 2), owing to the unequal leaf lobes and dark-colored gemmae, the species slightly resembles *Tritomaria exsectiformis* (Breidl.) Schiffn. ex Loeske and *T. mexicana* Bakalin but not *Lophozia*. The noted similarity is enhanced by the small cell size in the leaf, although they are not as elongated and not as clearly papillose-striolate as in the aforementioned *Tritomaria* taxa. However, the following features are immediately apparent:

1. Dark vinaceous-purple gemmae, which are not present in the aforementioned *Tritomaria*;
2. Unicellular gemmae (versus mostly bicellular, although in *T. mexicana*, they are also unicellular);
3. More equal leaf lobes and wider leaves.

Most likely, additional differences will be revealed upon the discovery of perianths. Another feature that brings *Lophozia vinacea* morphologically closer to *Tritomaria* is the leaves that sheath the stem at the base, as in most *Lophozia* and *Tritomaria*, but in the latter genus, the canal line makes a clear curve and is directed dorsally. The latter is more characteristic of *Tritomaria* and is rare in *Lophozia*. However, this feature is observed in *L. lacerata* and *L. dubia.* Notably, both latter species have colorless to greenish gemmae, and only *L. lacerata* sometimes produces brownish gemmae that make the differentiation from *Tritomaria* easy.

Within the *Lophozia koreana*–*L. dubia* clade, the new species is peculiar by the following:

1. Smaller cells varying within 15–20(–30) × 15–20(–25) μm (the distance from *L. koreana* in terms of cell size is less than that from *L. neglecta*);
2. Vinaceous-purple gemmae (not present in other taxa);
3. A stem in cross section with a distinct microcellous layer (a difference from *L. koreana* only);
4. Unequally lobed leaves (a difference only from *L. neglecta*);
5. Gemmae are located among the uppermost leaves, as is often the case in *Tritomaria* (but not in clusters at the tips of the lobes, as is often the case in *Lophozia* s. str., including *L. neglecta* and *L. koreana*). Among *Lophozia* s. str. taxa, this gemmae distribution is also characteristic of *L. fuscovirens*, where the gemmae are brown and the species’ geographic distribution is much more northern.

Another poorly studied species, *Lophozia nepalensis* Bakalin, should belong to the discussed *Lophozia koreana*–*L. dubia* clade morphologically. It is characterized by ascending shoots with abbreviated leaves, purple gemmae at the apex of the shoot, and a rusty-brownish plant color. Considering that purple coloration (and even hints of it in the form of a pinkish tint) is not known at all within *Lophozia* outside the discussed clade, this species likely belongs to this clade. Morphological similarity is greatest between *L. nepalensis* and *L. vinacea* (but not necessarily genetic similarity, since, for example, *L. koreana* is closer not to *L. neglecta* but rather to *L. vinacea* despite morphological differences). The distinguishing features between *L. vinacea* and *L. nepalensis* are as follows:

1. The gemmae are two-celled and 10–15 × 13–18 μm in *L. nepalensis* versus one-celled in *L. vinacea*, where they are only 10–12 μm in diameter;
2. Elongate shoots with gemmae are present in *L. nepalensis* but absent in *L. vinacea*;
3. The cells in the midleaf of *L. nepalensis* are 20–26 × 22–28 μm, whereas those in *L. vinacea* are 15–20(–30) × 15–20(–25) μm.

**Figure 10:**
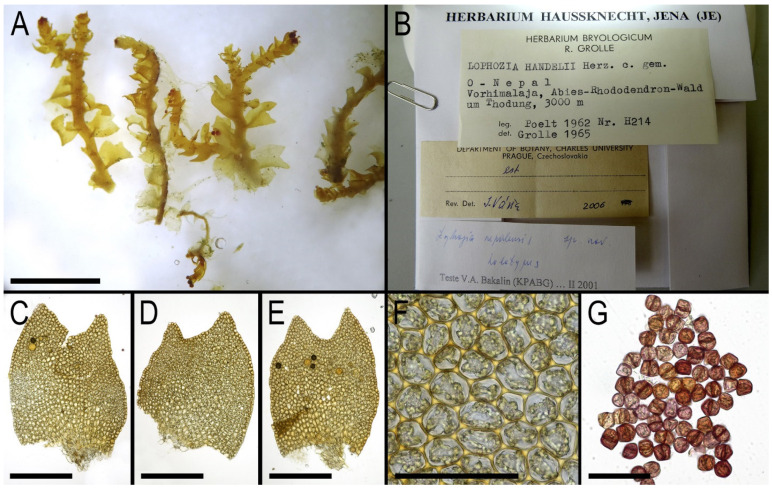
*Lophozia nepalensis* Bakalin: (**A**) shoots; (**B**) holotype label; (**C**–**E**) leaves; (**F**) midleaf cells; (**G**) gemmae. Scales: 2 mm for (**A**); 500 µm for (**C**–**E**); 100 µm for (**F**); 50 µm for (**G**). All from holotype Poelt H214 (JE).

The corresponding photographs of the holotype of *L. nepalensis* are provided in Figure 10.

### 2.3. Diversity of Lophozia in East Asia

The taxa treated above do not exhaust the diversity of *Lophozia* known in the East Asian floristic region [18] or the territorially equivalent East Asian floristic kingdom [19]. We list all of the *Lophozia* known there below:

*Lophozia ascendens* (Warnst.) R.M. Schust.—a boreal taxon of subcircumpolar distribution spreading southward to central Japan along the Pacific Ocean coast.

*Lophozia dubia* (=*L. pallida* (Steph.) Grolle)—a taxon with a mainly Sino-Himalayan distribution spreading southeastward to Indonesia and being the southernmost representative among broadly Asian *Lophozia*.

*Lophozia guttulata* (Lindb.) A. Evans—a taxon that was originally reviewed for Japan in East Asia under the name *Lophozia fauriana*—the later synonym of *L. guttulata*, although the genetic identity of Japanese and European populations has never been tested; it is also known in other parts of eastern East Asia to 35° N.

*Lophozia koreana*—a Korean–Japanese orohemiboreal rare taxon.

*Lophozia lacerata*—a poorly known Japanese taxon spreading northward to hemiarctic habitats along the Pacific Ocean; the genetic identity of northern and southern populations has never been tested.

*Lophozia lantratovae*—mainly East Asian taxon sparsely distributed across the area, although locally abundant, spreading westward to hemiboreal communities in the southern part of Siberia (Russia) and farther to the Caucasus.

*Lophozia neglecta*—strictly Sino-Himalayan hitherto known from the northern part of Yunnan Province only.

*Lophozia nepalensis*—strictly Sino-Himalayan species hitherto known from Nepal only.

*Lophozia silvicola* H. Buch—mainly a circumboreal taxon, although known from an isolated locality in Guizhou Province of China [20]; however, the material from the latter is so scarce that it prevents the testing of genetic identity and may actually belong to another taxon.

*Lophozia silvicoloides* N. Kitag.—mainly East Asian but widely spreading to the northern Holarctic in both the New and Old Worlds.

*Lophozia vinacea*—a strictly Sino-Himalayan taxon hitherto known from the northern part of Yunnan Province of China.

*Lophozia wenzelii* (Nees) Steph.—ambiguously reported from Japan by Kitagawa [21] who treated it as ‘atypical’ in some morphological features. Moreover, the habitat provided for the taxon in [21] (p. 284) is “moist sandy or muddy soil (rarely on humus) in the alpine zone, rarely in the subalpine zone”. The cited habitat seems unsuitable for true *L. wenzelii*, which grows in oligotrophic swamps. There are several possibilities regarding what Japanese ‘*L. wenzelii*’ may be, including *L. pacifica* Bakalin; *L. murmanica* Kaal.; and some varieties of *L. wenzelii* s.l., including its var. *lapponica* H. Buch et S.W. Arnell and var. *litoralis* (Arnell) Bakalin.

## 3. Discussion

Notably, all species in the group treated here are characterized by (1) purple coloration on the ventral side of the stem and the ventral bases of the leaves (features occurring in several other taxa of the genus), and (2) especially the gemmae, which can at least acquire a pink tint (with the exception of *L. dubia*)—a feature unknown in other groups in *Lophozia*. Another feature rarely found in *Lophozia* is the striolate leaf cuticle—a feature that develops very facultatively even in the present group and sometimes occurs outside of the group in *L. guttulata* (Lindb.) A. Evans. Unequally lobed leaves, occasionally striolate leaf cuticle, sometimes peculiarly curved leaves such as those in several *Tritomaria* taxa, and an ascending growth habit emphasize the similarity to *Tritomaria* and, perhaps, indicate a substantial group of species that evolved similar morphotypes in the Sino-Himalaya and adjacent areas. With the exception of the Korean–Japanese *L. koreana*, all of them are found in the Sino-Himalaya, although they are also found outside the latter area (especially as *L. dubia*, cf. [3]. This group might have been more widespread earlier. Regardless, this group did not achieve wide distribution in comparison with the group of green-gemmous *Lophozia*, which ‘filled’ the entire northern Holarctic ecosystem with possibly young, morphologically poorly distinguishable species in a broad sense referable to the *L. ventricosa* (Dicks.) Dumort. species complex [4]. The only green-gemmous species within the group is *L. dubia*. It was described at least three times under different names, including *L. handelii* Herzog and *Anastrophyllum pallidum* Steph. cf. [2,3]. All the taxa of the group (*L. koreana*, *L. dubia*, *L. neglecta*, *L. vinacea*, and possibly *L. nepalensis*) may illustrate an alternative lineage in the evolution of *Lophozia*, which is now present in East Asia and is particularly diverse in the Sino-Himalaya.

It is possible to assume that various phylogenetic lines undertook similar attempts to colonize the vast landscapes in the North Holarctic. These landscapes—vast treeless areas in the mountains and the north—formed as a result of steady cooling evident from the second half of the Miocene. While some lineages achieved obvious success (many examples exist from the last deglaciation event in several plant groups, e.g., [22]), others persisted only in extreme isolation. Within *Lophozia*, as shown earlier [3], several clades may be distinguished. One can be called predominantly East Asian, and the others can be called predominantly Northern Holarctic. In Figure 1 and Figure 2, the former is the uppermost clade from *Lophozia koreana* to *L. silvicoloides* or *L. neglecta*. In the northern Holarctic, the greatest abundance was achieved by the green-gemmous *Lophozia* species. Many taxa in the latter group are probably quite young, such that morphological features between species are often unclear, as outlined in the Introduction following the citation from Schuster [4]. The East Asian group is represented by species that are more southern in distribution, with more clearly defined morphological features. Within the ‘East Asian clade’, species with colored gemmae are common, whereas this feature is generally uncommon within the genus, although with notable exceptions for the taxa with brown-colored gemmae: *L. lantratovae* and *L. fuscovirens*. These mountain East Asian taxa have been virtually unable to spread beyond this floristic region or (alternatively) have generally gone extinct outside the zone. However, within East Asia, these species may constitute the main diversity of the genus *Lophozia*. Considering the insufficiency of data on *Lophozia* from the Sino-Himalaya, the three species found only there most likely indicate a potentially high diversity of the genus, which is still far from fully understood.

As follows from the Section 2, there are 12 species in total occurring in East Asia, although the taxonomic identity is unclear in some cases. Of these, four have their main range in the northern Holarctic in Asia, penetrating further south, along the amphi-Pacific areas; two are East–East Asian; one is widely East Asian and reaching the Caucasus to the west; one is East–East Asian and widely penetrating the northern Holarctic; and four are Sino-Himalayan. Several of these taxa have been described or reviewed within the past few decades, and we expect that the Sino-Himalayan bulk of *Lophozia* taxa may still be poorly understood and that new findings are likely.

## 4. Materials and Methods

### 4.1. Specimen Collection

All the specimens included in this study for morphological analysis were collected at a notable location: Shangri-La County in the Diqing Tibetan Autonomous Prefecture of Yunnan Province, China. This area is surrounded by Sichuan Province on all sides except the south and is characterized by high mountainous terrain. Our specimens were collected near 4000 m a.s.l. At these altitudes, in the study area, the vegetation possesses a transitional character from that of dark coniferous forests (with mainly *Abies* Mill. dominating) to tree *Rhododendron* stands. The studied communities are developed on ancient limestone deposits, which partly influences the vegetation characteristics and did not affect our findings, since all of them were made on decaying wood, not on soil. According to the Köppen–Geiger climate classification [23,24], the collection sites have a monsoon-influenced subarctic climate (Dwc) with dry winters and cold summers. The annual mean temperature varies between 3.3 and 2.9 °C, and the annual precipitation is approximately 724–753 mm; the warmest quarter is the wettest in areas with a mean temperature ranging from 9.4 to 10.2 °C and precipitation ranging from 339 to 365 mm per quarter [25,26]. During sampling, each specimen was assigned a unique field number, geographic coordinates, altitude, moisture condition, shading, substrate, and community type. The specimens were delivered alive to the Laboratory of Cryptogamic Biota (herbarium acronym VBGI), where they were examined for morphology. The possibility that putative new species had been collected was suggested during the initial microscopic examination. Individual plants were subsequently isolated from the patch and dried in plastic bags with silica gel for molecular genetic analysis, as described below.

### 4.2. Specimen Analysis

#### 4.2.1. Taxon Sampling

Sequences for the *Lophozia dubia* specimen voucher C-83-7-18 (*trn*G, *rps*4, *rbc*L), *Lophozia* sp.1 vouchers C-86-1-18 and C-86-2-18 (ITS1-2, *trn*L-F, *trn*G, *rps*4, *rbc*L), and *Lophozia* sp.2 vouchers C-83-24a-18 and C-83-26-18 (ITS1-2, *trn*L-F, *trn*G, *rps*4, *rbc*L) from China (Yunnan Province); *Lophozia fuscovirens* voucher K-52-9-22 (ITS1-2, *trn*L-F, *trn*G, *rps*4, *rbc*L) and *Lophozia pacifica* voucher K-107-38-21 (ITS1-2, *trn*L-F) from Russia (Russian Far East); and *Lophozia koreana* vouchers Kor-74-5-19, Kor-75-15-19, Kor-76-1-19 (*rps*4, *rbc*L) from South Korea (herbarium specimens of all listed species are available in VBGI) were obtained by the authors, and nucleotide data for 159 specimens were downloaded from the National Center for Biotechnology Information (NCBI) GenBank. DNA vouchers, including GenBank accession numbers and voucher details, are listed in Table A1 in Appendix A. When compiling the dataset, we were guided by the fact that at least one of the potentially new species has some morphological similarities to Tritomaria, and therefore, we present an expanded dataset, including many related groups.

Phylogenies were constructed for the Lophoziaceae species from several closely related sections and some species from the families Scapaniaceae and Anastrophyllaceae, with sequences available in GenBank for the involved loci used in this account preparation. We tried to select the sequences where data on all loci used here were based on the same specimen. This was necessary for the subsequent construction of consensus trees. Specimens of *Cephalozia bicuspidata* (L.) Dumort. and *Fuscocephaloziopsis affinis* (Lindb. ex Steph.) Váňa et L. Söderstr. (Cephaloziaceae Mig.) were used as an outgroup for tree rooting. The choice of outgroup was based on previous studies by Heinrichs et al. [27], Feldberg et al. [28], and Konstantinova et al. [29].

#### 4.2.2. DNA Isolation, Amplification, and Sequencing

DNA was extracted from dried liverwort tissues using the HiPure SF Plant DNA Kit (Guangzhou Magen Biotechnology Co., Ltd. (Magen), Guangzhou, China), following the manufacturer’s protocols. Amplification of ITS1–2, *trn*L–F, *trn*T-*trn*F, *trn*G-intron, *rb*cL, and *rps*4 was performed using an Encyclo Plus PCR Kit (Evrogen, Moscow, Russia) with the primers listed in Table 4.

The polymerase chain reaction was performed in a total volume of 20 µL, including 1 µL of template DNA, 0.4 µL of 50× Encyclo polymerase Mix (concentration in the final volume of the reaction mixture was 1×), 4 µL of 5× Encyclo Red buffer (concentration in the final volume of the reaction mixture was 1×), 0.4 µL of dNTP mixture (0.16 mM each), 13.4 µL (for *trn*L–F, *trn*T–*trn*F, *trn*G–intron, *rbc*L, and *rps*4) or 12.4 µL (for ITS1–2) of nuclease-free deionized water, 1 µL of dimethylsulfoxide/DMSO (5% in the final volume of the reaction mixture) for ITS1–ITS2 nrDNA, and 0.4 µL of each primer (forward and reverse, 0.2 µM in the final volume of the reaction mixture). The concentration of Mg^2+^ in the final reaction was 3.5 mM. All reagents, excluding DMSO, were obtained from the Encyclo Plus PCR Kit, Evrogen, Moscow, Russia.

Polymerase chain reactions were performed using the protocols for amplification listed in Table 5. In some cases, where the *trn*L-F locus was part of the *trn*T-*trn*F locus, the protocol was as in Table 6.

#### 4.2.3. Phylogenetic Analyses

**Figure A1:**
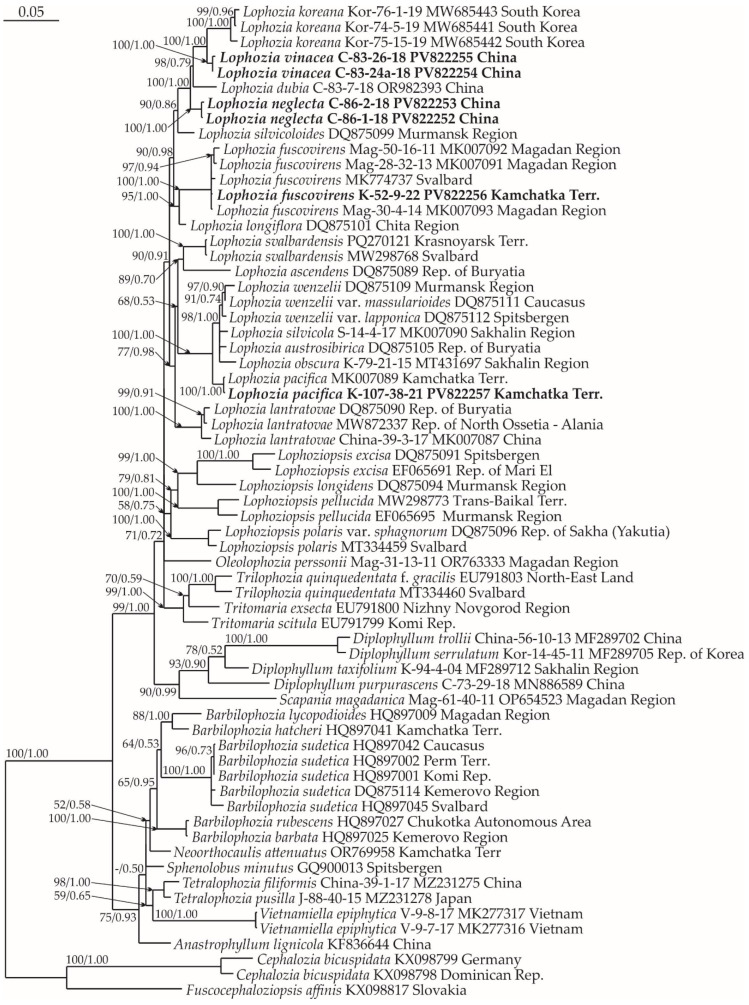
Phylogram obtained from a Bayesian analysis of the Lophoziaceae species and related taxa based on ITS1-2 nrDNA. Newly obtained species are marked in bold. Bootstrap support values > 50% in ML analysis and Bayesian posterior probabilities PP > 0.50 are indicated. Scale bar denotes the number of nucleotide substitutions per site.

**Figure A2:**
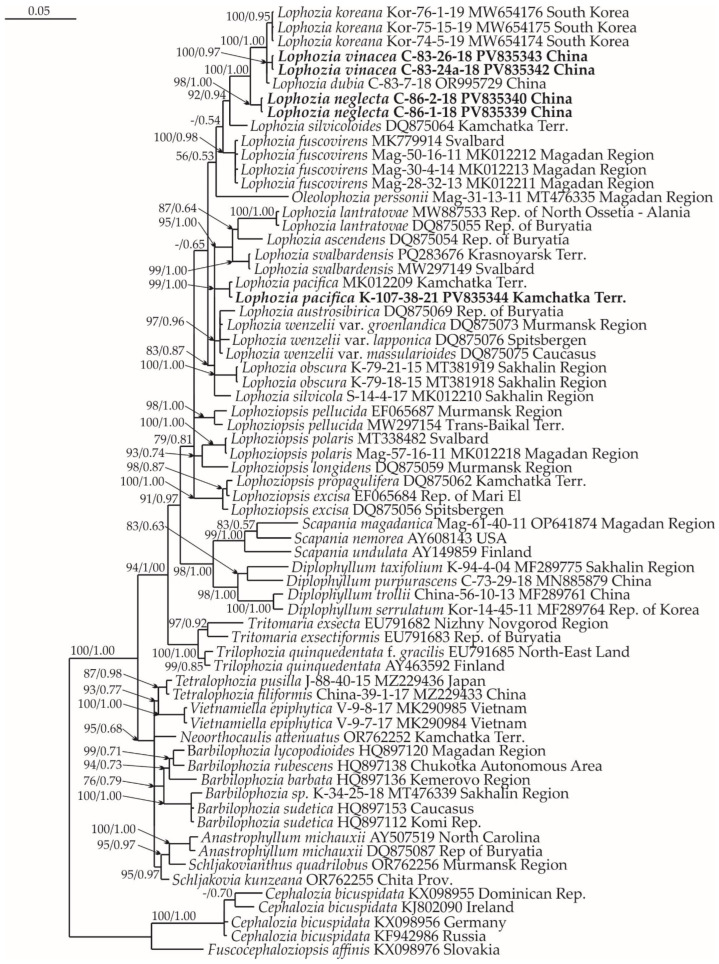
Phylogram obtained from a Bayesian analysis of the Lophoziaceae species and related taxa based on *trn*L-*trn*F cpDNA. Newly obtained species are marked in bold. Bootstrap support values > 50% in ML analysis and Bayesian posterior probabilities PP > 0.50 are indicated. Scale bar denotes the number of nucleotide substitutions per site.

**Figure A3:**
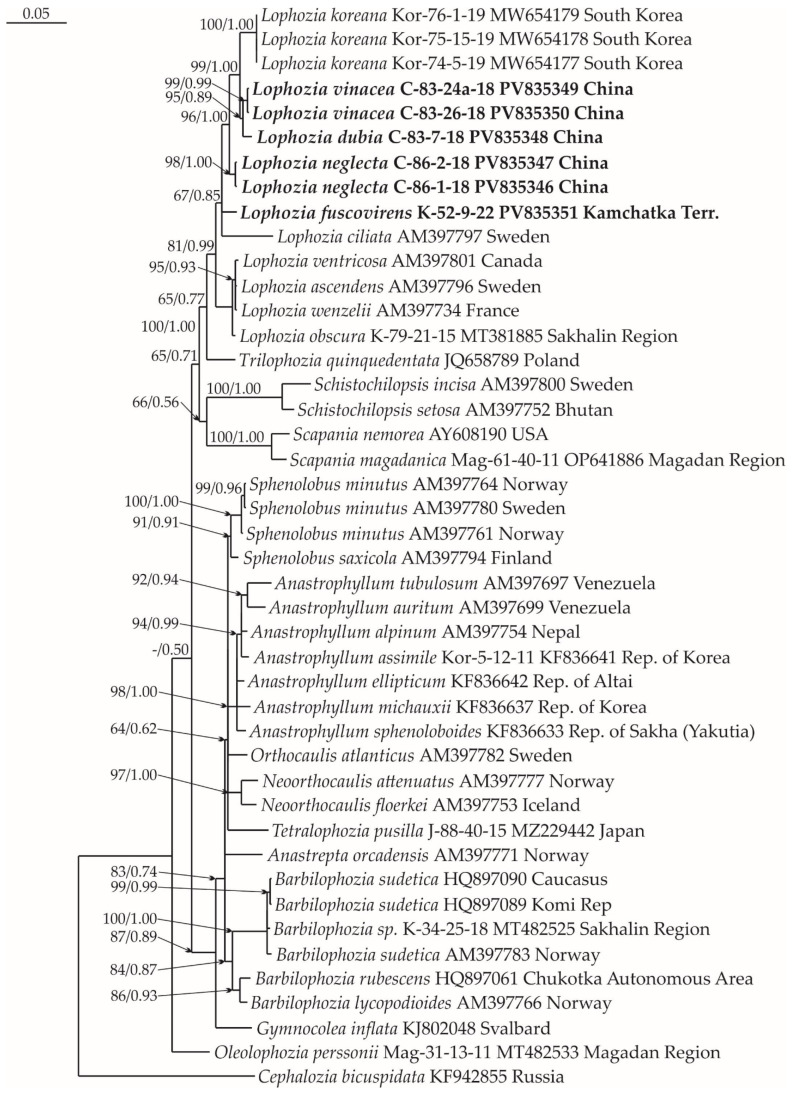
Phylogram obtained from a Bayesian analysis of the Lophoziaceae species and related taxa based on *trn*G-intron cpDNA. Newly obtained species are marked in bold. Bootstrap support values > 50% in ML analysis and Bayesian posterior probabilities PP > 0.50 are indicated. Scale bar denotes the number of nucleotide substitutions per site.

**Figure A4:**
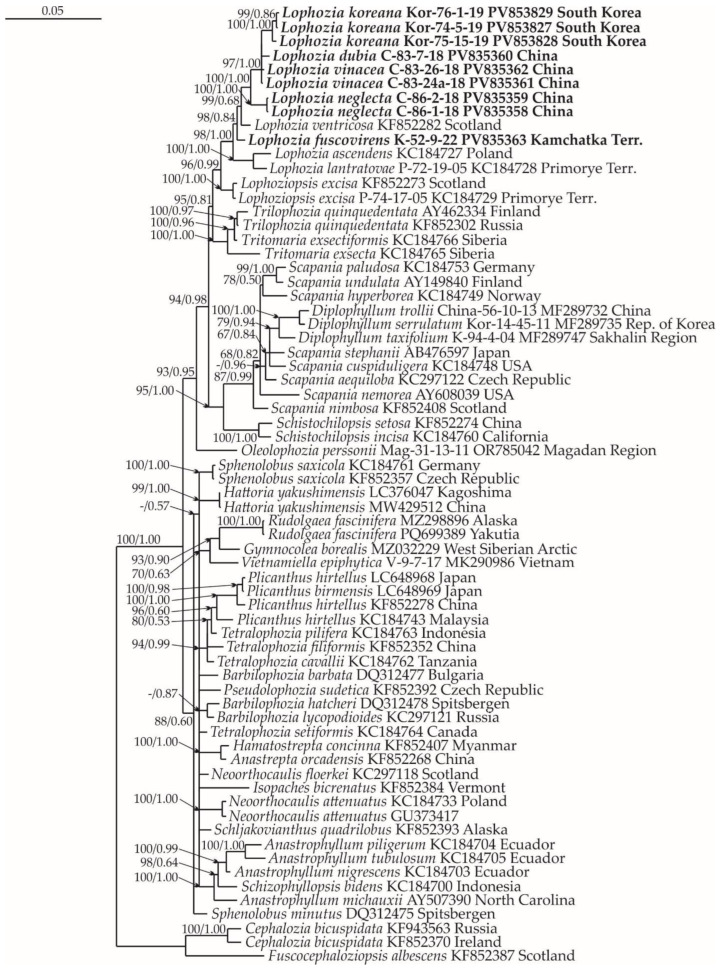
Phylogram obtained from a Bayesian analysis of the Lophoziaceae species and related taxa based on *rbc*L cpDNA. Newly obtained species are marked in bold. Bootstrap support values > 50% in ML analysis and Bayesian posterior probabilities PP > 0.50 are indicated. Scale bar denotes the number of nucleotide substitutions per site.

**Figure A5:**
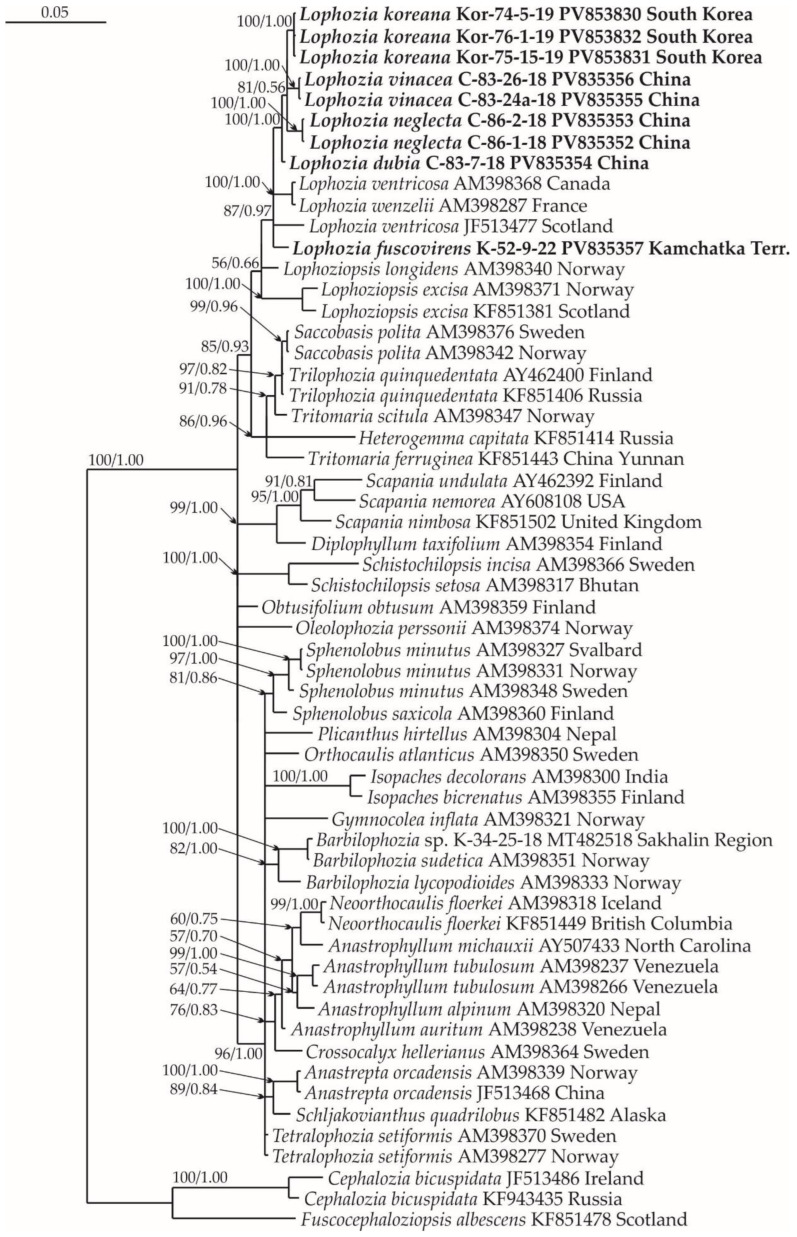
Phylogram obtained from a Bayesian analysis of the Lophoziaceae species and related taxa based on *rps*4 cpDNA. Newly obtained species are marked in bold. Bootstrap support values > 50% in ML analysis and Bayesian posterior probabilities PP > 0.50 are indicated. Scale bar denotes the number of nucleotide substitutions per site.

Four datasets were compiled for individual loci of the chloroplast genome, namely, trnL-F, trnG, rbcL, and rps4, along with one dataset for the nuclear genome, namely, ITS1-2. A preliminary comparative analysis of the obtained phylogenetic trees for each locus revealed their congruence. However, despite high congruence, the vouchers for each locus were commonly different from the vouchers for other loci. As a result, we could not compile a joint alignment that would include all treated loci for further analysis. The latter limitation led us to use three combined trees for the following combinations: ITS-trnL-F (Figure 1), trnG-rps4 (Figure 2), and rbcL-rps4 (Figure 3). The combination of loci into three pairs was based on the overlap of available data from different loci. For example, ITS 1, 2 was combined with trnL-F, since many samples had data from both of these loci, etc. However, all trees for each locus separately are provided in Appendix B (Figure A1, Figure A2, Figure A3, Figure A4 and Figure A5). All original datasets were aligned using MAFFT [36,37,38], with standard settings, and then manually edited in BioEdit ver. 7.2.5 [39]. All positions of the final alignment were included in the phylogenetic analyses. Missing data at the ends of the regions and gaps were treated as missing data.

Phylogenetic trees were reconstructed using two approaches: maximum likelihood (ML) [40] with IQ-tree ver. 2.2.2.6 [41] and Bayesian inference (BI) [42] with MrBayes ver. 3.2.7 [43]. For the ML analysis, the best-fitting evolutionary model of nucleotide substitutions according to the Bayesian information criterion (BIC) value differed for every single alignment: for ITS1–ITS2, it was TIM+F+I+G4; for the *trn*L–*trn*F alignment—HKY+F+I+G4; for the *trn*G—K3Pu+F+G4; for the *rbc*L and combined dataset *rbc*L + *rps*4—TIM3+F+I+R2; for the *rps*4—TVM+F+I+G4; for the combined ITS1–2 + *trn*L–F—TIM3e+I+G4; and for the combined *trn*G + *rps*4—K3Pu+F+I+R3. Each of these models was determined by ModelFinder (a model selection method implemented in IQ-tree) [44]. Consensus trees were constructed with 1000 bootstrap replicates. Bootstrap support (BS) percentage values were calculated.

BI analyses were performed by running two parallel analyses via the GTR+I+G model. The analysis consisted of four Markov chains. Chains were run for five million generations, and trees were sampled every 500th generation. The first 2500 trees in each run were discarded as burn-in; thereafter, 15,000 trees were sampled from both runs to produce the resulting tree. Bayesian posterior probabilities were calculated from the trees sampled after burn-in. The average standard deviation of the split frequencies between two runs before the analysis was stopped was 0.003 for ITS1–ITS2, *trn*G, and ITS1–2 + *trn*L–F; for *trn*L–*trn*F, it was 0.005; for *rbc*L, *rps*4*,* and *rbc*L + *rps*4, it was 0.004; and for *trn*G + *rps*4, it was 0.002.

The infraspecific and interspecific variation for the species *Lophozia koreana, Lophozia neglecta, Lophozia vinacea, Lophozia dubia*, and *Lophozia fuscovirens* of all eight datasets were quantified as the average pairwise *p*-distances calculated in Mega XII [45] using the pairwise deletion option for counting gaps, the proportion of nucleotide sites that are different (d: Transitions + Transversions), and 1000 bootstrap replicates.

## 5. Conclusions

The newly described taxa of *Lophozia* (*L. neglecta* and *L. vinacea*) add to the understanding of the diversity of the genus in Sino-Himalaya and possess distinctive features, including the presence of purple or pinkish pigmentation of the gemmae. Not all species known from East Asia are found further north, and conversely, North Holarctic taxa do not always penetrate East Asia. Despite the limited available data, East Asia, especially the Sino-Himalayan region, has pronounced species specificity, which is likely to increase with further research.

## Figures and Tables

**Table 1:** The characteristics of the tested nucleotide sequence alignments according to Table A1.

Locus	Number of Sequences in the Alignment	Total Sites in the Alignment	Conservative Sites		Variable Sites		Parsimony-Informative Sites		Base Frequencies			
			Base Pairs	%	Base Pairs	%	Base Pairs	%	A	C	G	T
ITS1–ITS2	65	890	415	46.63	453	50.90	341	38.31	0.174	0.291	0.338	0.197
*trn*L–*trn*F	68	484	288	59.50	177	36.57	142	29.34	0.347	0.152	0.173	0.328
*trn*G	44	640	352	55.00	255	39.84	139	21.72	0.363	0.134	0.127	0.376
*rbc*L	69	1131	843	74.54	288	25.46	201	17.77	0.289	0.172	0.218	0.322
*rps*4	58	741	468	63.16	273	36.84	186	25.10	0.358	0.149	0.174	0.320
ITS1–2 + *trn*L–F	65	1367	686	50.18	631	46.16	481	35.19	0.237	0.241	0.278	0.245
*trn*G + *rps*4	44	1253	753	60.10	463	36.95	266	21.23	0.360	0.143	0.152	0.346
*rbc*L + *rps*4	34	1842	1333	72.37	507	27.52	331	17.97	0.317	0.162	0.201	0.320

**Table 2:** The log-likelihood in the ML analysis and in Bayesian analysis (arithmetic mean) of the tested nucleotide sequence alignments.

Locus	ML Analysis	Bayesian Analysis	
		Run 1	Run 2
ITS1–ITS2	−7467.54	−7524.67	−7523.95
*trn*L–*trn*F	−3199.90	−3265.75	−3264.95
*trn*G	−3325.28	−3362.99	−3362.03
*rbc*L	−5521.40	−5600.09	−5603.52
*rps*4	−3988.17	−4048.25	−4044.00
ITS1–2 + *trn*L–F	−11,246.90	−11,293.19	−11,297.05
*trn*G + *rps*4	−6694.954	−6755.03	−6756.83
*rbc*L + *rps*4	−7840.28	−7876.37	−7878.80

**Table 3:** Infraspecific and interspecific *p*-distances. Calculation based on ITS1-2/*trn*L-F/*trn*G/*rbc*L/*rps*4 nucleotide sequence data. The number of base differences per site from averaging over all sequence pairs within and between each group is shown; “n/c”—not calculated due to the presence of a single specimen; newly obtained sequences and measured *p*-Distances are bolded.

№	Taxa	Infraspecific *p*-Distances, ITS1–ITS2/*trn*L–*trn*F/*trn*G/*rbc*L/*rps*4, %	Interspecific *p*-Distances, ITS1–ITS2/*trn*L–*trn*F/*trn*G/*rbc*L/*rps*4, %			
			1	2	3	4
1	*Lophozia koreana*	0.52/0/0/0.18/0				
**2**	***Lophozia vinacea***	**0.26/0/0/0.11/0**	**2.8/0.45/1.57/0.76/0.85**			
3	*Lophozia dubia*	n/c/n/c/n/c/n/c/n/c	5.10/0.26/2.08/1.02/0.42	**3.48/0.25/0.87/0.38/0.69**		
**4**	***Lophozia neglecta***	**0.13/0/0/0/0**	**4.19/2.05/2.60/1.61/1.13**	**2.79/2.04/1.57/1.29/1.38**	**3.17/2.04/2.08/1.37/0.97**	
5	*Lophozia fuscovirens*	0.41/0/n/c/n/c/n/c	7.09/3.9/3.84/1.82/1.56	**5.67/3.89/2.46/1.14/1.8**	6.32/3.34/2.97/1.58/1.11	**5.22/3.20/2.27/1.44/1.81**

**Table 4:** Primers used in polymerase chain reaction (PCR) and cycle sequencing.

Locus	Sequence (5′-3′)	Direction	Annealing Temperature (°C)	Reference
*trn*L–*trn*F cpDNA	CGAAATTGGTAGACGCTGCG	forward	62	[17]
*trn*L–*trn*F cpDNA	TGCCAGAAACCAGATTTGAAC	reverse	58	[17]
*trn*T–F cpDNA	GAGGTCCTCGATAACGNGACATAA	forward	70–72	[30]
ITS1–ITS2 nrDNA	ACCTGCGGAAGGATCATTG	forward	58	[31]
ITS1–ITS2 nrDNA	GATATGCTTAAACTCAGCGG	reverse	58	[32]
ITS1–ITS2 nrDNA	CGTTGTGAGAAGTTCATTAAACC	reverse	64	[33]
*trn*G-intron cpDNA	CGGGTAGCGGGAATCGAAC	forward	62	present study
*trn*G-intron cpDNA	GCGGGTATAGTTTAGTGG	reverse	54	[34]
*rbc*L cpDNA	ATGTCACCACAAACAGAGACTAAAGC	forward	50	[35]
*rbc*L cpDNA	GCAGCAGCTAAMTCRGGACTCCA	reverse	50	[30]
*rps*4 cpDNA	GATGGTTGAGTGGTYTAAGAT	forward	58–60	present study
*rps*4 cpDNA	ATGTCCCGTTATCGAGG	reverse	52	present study

**Table 5:** The protocols used in PCR for ITS1-2, *trn*L-F, *trn*G-intron, *rbc*L and *rps*4 loci.

Step	Time	Temperature, °C	Cycles
Initial denaturation	3 min	95 (ITS1-2, *trn*L-F, *trn*G-intron), 94 (*rbc*L, *rps*4)	35–40
Denaturation	30 s (ITS1-2, *trn*L-F, *trn*G-intron), 40 s (*rbc*L, *rps*4)	94	
Annealing	20 s (*trn*L-F), 30 s (ITS1-2, *trn*G-intron), 90 s (*rbc*L, *rps*4)	50 (*rbc*L, *rps*4), 56 (*trn*G-intron), 58 (ITS1-2, *trn*L-F)	
Elongation	30 s (ITS1-2, *trn*L-F, *trn*G-intron), 2 min (*rbc*L, *rps*4)	72	
Final elongation	5 min	72	1

**Table 6:** The protocol used in PCR for *trn*T-F locus.

Step	Time	Temperature, °C	Cycles
Initial denaturation	3 min	95	35
Denaturation	30 s	95	
Annealing	70 s	60	
Elongation	90 s	72	
Final elongation	5 min	72	1

## Data Availability

The original contributions presented in this study are included in this article. Further inquiries can be directed to the corresponding authors.

## Appendix A

**Table A1:** The list of voucher details and GenBank accession numbers for the specimens used in the phylogenetic analysis in the present paper. The newly obtained sequences are marked in bold.

#	GenBank Name	Accepted Name	Specimen Voucher	ITS1-2 nrDNA	*trn*L-F cpDNA	*trn*G	*rbc*L	*rps*4
1	*Anastrepta orcadensis* (Hook.) Schiffn.	*Anastrepta orcadensis* (Hook.) Schiffn.	China, Yunnan, Long D.G. 34711 (E)				KF852268	JF513468
2	*Anastrepta orcadensis* (Hook.) Schiffn.	*Anastrepta orcadensis* (Hook.) Schiffn.	Norway, L. Soederstroem, Soederstroem 2003/017 (BOL)			AM397771		AM398339
3	*Anastrophyllum alpinum* Steph.	*Anastrophyllum alpinum* Steph.	Nepal, Long 30460 (E)			AM397754		AM398320
4	*Anastrophyllum assimile* (Mitt.) Steph.	*Anastrophyllum assimile* (Mitt.) Steph.	South Korea, V.A. Bakalin, Kor-5-12-11 (VBGI)			KF836641		
5	*Anastrophyllum auritum* (Lehm.) Steph.	*Anastrophyllum auritum* (Lehm.) Steph.	Venezuela, L. Soederstroem, Soederstroem 2004/065 (BOL)			AM397699		AM398238
6	*Anastrophyllum bidens* (Reinw., Blume et Nees) Steph.	*Schizophyllopsis bidens* (Reinw., Blume et Nees) Váňa et L. Söderstr.	Indonesia, Gradstein 12067 (GOET)				KC184700	
7	*Anastrophyllum ellipticum* Inoue	*Anastrophyllum ellipticum* Inoue	Russia, Altai Territory, Yu. Mamontov, 330/2 (KPABG)			KF836642		
8	*Anastrophyllum* *lignicola* D.B. Schill et D.G. Long	*Anastrophyllum ellipticum* Inoue	China, D. Long, 24067 (KPABG)	KF836644				
9	*Anastrophyllum michauxii* (F. Weber) H. Buch	*Anastrophyllum michauxii* (F. Weber) H. Buch	USA, North Carolina, Clingman’s Dome, M. Sargent s n (ABSH)		AY507519		AY507390	AY507433
10	*Anastrophyllum michauxii* (F. Weber) H. Buch	*Anastrophyllum michauxii* (F. Weber) H. Buch	South Korea, S.-S. Choi, Hepaticae Korea Exsiccatae F.II #54, 115520 (KPABG)			KF836637		
11	*Anastrophyllum minutum* (Schreb.) R.M. Schust.	*Sphenolobus minutus* (Schreb.) Berggr.	Norway, Soederstroem 2004/271 (BOL)			AM397764		AM398331
12	*Anastrophyllum minutum* (Schreb.) R.M. Schust.	*Sphenolobus minutus* (Schreb.) Berggr.	Norway, Svalbard, L. Soederstroem, Soederstroem 2004/327 (BOL)			AM397761		AM398327
13	*Anastrophyllum minutum* (Schreb.) R.M. Schust. var. *minutum*	*Sphenolobus minutus* (Schreb.) Berggr.	Sweden, L. Soederstroem et P. Manyanga, Soederstroem 2003/054 (BOL)			AM397780		AM398348
14	*Anastrophyllum nigrescens* (Mitt.) Steph.	*Anastrophyllum nigrescens* (Mitt.) Steph.	Ecuador, Schaefer-Verwimp et al. 24444 (GOET)				KC184703	
15	*Anastrophyllum piligerum* (Nees) Steph.	*Anastrophyllum piligerum* (Nees) Steph.	Ecuador, Schaefer-Verwimp et al. 24271 (GOET)				KC184704	
16	*Anastrophyllum* *saxicola* (Schrad.) R.M. Schust.	*Sphenolobus saxicola* (Schrad.) Steph.	Finland, L. Soederstroem & P. Manyanga, Soederstroem 2003/099 (BOL)			AM397794		AM398360
17	*Anastrophyllum sphenoloboides* R.M. Schust.	*Schizophyllopsis sphenoloboides* (R.M. Schust.) Váňa et L. Söderstr.	Russia, Yakutiya Rep., V.A. Bakalin, 101592 (KPABG)			KF836633		
18	*Anastrophyllum tubulosum* (Nees) Grolle	*Anastrophyllum tubulosum* (Nees) Grolle	Ecuador, Schaefer-Verwimp et al. 24464 (GOET)				KC184705	
19	*Anastrophyllum tubulosum* (Nees) Grolle	*Anastrophyllum tubulosum* (Nees) Grolle	Venezuela, L. Soederstroem, Soederstroem 2004/030 (BOL)			AM397697		AM398237
20	*Anastrophyllum tubulosum* (Nees) Grolle	*Anastrophyllum tubulosum* (Nees) Grolle	Venezuela, L. Soederstroem, Soederstroem 2004/120 (BOL)					AM398266
21	*Barbilophozia attenuata* (Mart.) Loeske	*Neoorthocaulis* *attenuatus* (Mart.) L. Söderstr., De Roo et Hedd.	Norway, L. Soederstroem, Soederstroem 2003/020 (BOL)			AM397777		
22	*Barbilophozia attenuata* (Mart.) Loeske	*Neoorthocaulis* *attenuatus* (Mart.) L. Söderstr., De Roo et Hedd.	4230531 (H)				GU373417	
23	*Barbilophozia atlantica* (Kaal.) Müll. Frib.	*Orthocaulis atlanticus* (Kaal.) H. Buch	Sweden, L. Soederstroem & P. Manyanga, Soederstroem 2003/057 (BOL)					AM398350
24	*Barbilophozia barbata* (Schmidel ex Schreb.) Loeske	*Barbilophozia barbata* (Schmidel ex Schreb.) Loeske	Russia, Kemerovskaya Prov., N.A. Konstantinova, 122-1-00 (KPABG)	HQ897025	HQ897136			
25	*Barbilophozia barbata* (Schmidel ex Schreb.) Loeske	*Barbilophozia barbata* (Schmidel ex Schreb.) Loeske	Bulgaria, Hentschel Bryo 0753 (GOET)				DQ312477	
26	*Barbilophozia floerkei* (F. Weber et D. Mohr) Loeske	*Neoorthocaulis floerkei* (F. Weber et D. Mohr) L. Söderstr., De Roo et Hedd.	Iceland, A. Seneca & L. Soederstroem, Soederstroem 2004/457 (BOL)			AM397753		AM398318
27	*Barbilophozia hatcheri* (A. Evans) Loeske	*Barbilophozia hatcheri* (A. Evans) Loeske	Russia, Commander Island, V.A. Bakalin, K-1-2-02-VB (KPABG)	HQ897041				
28	*Barbilophozia hatcheri* (A. Evans) Loeske	*Barbilophozia hatcheri* (A. Evans) Loeske	Norway, Spitsbergen, Hentschel Bryo 0492				DQ312478	
29	*Barbilophozia lycopodioides* (Wallr.) Loeske	*Barbilophozia lycopodioides* (Wallr.) Loeske	Russia, Magadanskaya Prov., O. Mochalova, 106872 (KPABG)	HQ897009	HQ897120			
30	*Barbilophozia lycopodioides* (Wallr.) Loeske	*Barbilophozia lycopodioides* (Wallr.) Loeske	Russia, N.A. Konstantinova 20 September 1994 (F)				KC297121	
31	*Barbilophozia lycopodioides* (Wallr.) Loeske	*Barbilophozia lycopodioides* (Wallr.) Loeske	Norway, L. Soederstroem, Soederstroem 2003/019 (BOL)			AM397766		AM398333
32	*Barbilophozia rubescens* (R.M. Schust. et Damsh.) Kartt. et L. Söderstr.	*Barbilophozia rubescens* (R.M. Schust. et Damsh.) Kartt. et L. Söderstr.	Russia, Chukotka A.O., E. Kuzmina, 100171 (KPABG)	HQ897027	HQ897138	HQ897061		
33	*Barbilophozia sudetica* (Nees ex Huebener) L. Söderstr., De Roo et Hedd.	*Pseudolophozia sudetica* (Nees ex Huebener) Konstant. et Vilnet	Czech Republic, B. Shaw 12991 (DUKE)				KF852392	
34	*Cephalozia bicuspidata* (L.) Dumort.	*Cephalozia bicuspidata* (L.) Dumort.	Dominican Republic, Province San Jose de Ocoa, Schaefer-Verwimp et Verwimp 26879/A (M)	KX098798	KX098955			
35	*Cephalozia bicuspidata* (L.) Dumort.	*Cephalozia bicuspidata* (L.) Dumort.	Germany, Baden-Wuerttemberg, Schaefer-Verwimp et Verwimp 27426 (M)	KX098799	KX098956			
36	*Cephalozia bicuspidata* (L.) Dumort.	*Cephalozia bicuspidata* (L.) Dumort.	Russia, N.A. Konstantinova (F)		KF942986	KF942855	KF943563	KF943435
37	*Cephalozia bicuspidata* (L.) Dumort.	*Cephalozia bicuspidata* (L.) Dumort.	Ireland, Stotler et C.-Stotler s.n. (ABSH)		KJ802090		KF852370	JF513486
38	*Anastrophyllum hellerianum* (Nees ex Lindenb.) R.M. Schust.	*Crossocalyx hellerianus* (Nees ex Lindenb.) Meyl.	Sweden, Soederstroem 2003/081 (BOL)					AM398364
39	*Diplophyllum purpurascens* Bakalin et Vilnet	*Diplophyllum purpurascens* Bakalin et Vilnet	China, Yunnan Province, Dali Prefecture, V.A. Bakalin & W.Z. Ma, C-73-29-18	MN886589	MN885879			
40	*Diplophyllum* *serrulatum* (Müll. Frib.) Steph.	*Diplophyllum* *serrulatum* (Müll. Frib.) Steph.	Republic of Korea, Jeonnam Province, V.A. Bakalin, Kor-14-45-11	MF289705	MF289764		MF289735	
41	*Diplophyllum taxifolium* (Wahlenb.) Dumort.	*Diplophyllum taxifolium* (Wahlenb.) Dumort.	Russia, Sakhalin Prov., Kuril Islands, Paramushir Isl., V.A. Bakalin, K-94-4-04	MF289712	MF289775		MF289747	
42	*Diplophyllum taxifolium* (Wahlenb.) Dumort.	*Diplophyllum taxifolium* (Wahlenb.) Dumort.	Finland, Soederstroem 2003/098 (BOL)					AM398354
43	*Diplophyllum trollii* Grolle	*Diplophyllum trollii* Grolle	China, Guizhou Prov., V.A. Bakalin, China-56-10-13 (VBGI)	MF289702	MF289761		MF289732	
44	*Fuscocephaloziopsis* *affinis* (Lindb. ex Steph.) Váňa et L. Söderstr.	*Fuscocephaloziopsis* *affinis* (Lindb. ex Steph.) Váňa et L. Söderstr.	Slovakia, Banskobystricky Kraj, Vana 6 (Vana)	KX098817	KX098976			
45	*Fuscocephaloziopsis* *albescens* (Hook.) Váňa et L. Söderstr.	*Fuscocephaloziopsis* *albescens* (Hook.) Váňa et L. Söderstr.	United Kingdom, Banff, Scotland, D.G. Long & G.P. Rothero 36976 (E)				KF852387	KF851478
46	*Gymnocolea borealis* (Frisvoll et Moen) R.M. Schust.	*Rudolgaea borealis* (Frisvoll et Moen) Potemkin et Vilnet	Russia, West Siberian Arctic, Gydansky Peninsula, vicinities of Yambuto Lake, E.I. Troeva, G1-138 (LE)				MZ032229	
47	*Rudolgaea fascinifera* (Potemkin) Potemkin et Vilnet	*Rudolgaea fascinifera* (Potemkin) Potemkin et Vilnet	Russia, Republic of Sakha, Yakutia, Kytalyk National Park, E.D. Lapshina, 019E-6-23 126373 (KPABG)				PQ699389	
48	*Gymnocolea fascinifera* Potemkin	*Rudolgaea fascinifera* (Potemkin) Potemkin et Vilnet	USA, Alaska, A.D. Potemkin, 92-9701 (LE)				MZ298896	
49	*Gymnocolea inflata* (Huds.) Dumort.	*Gymnocolea inflata* (Huds.) Dumort.	Svalbard, Spitsbergen, N.A. Konstantinova & A.N. Savchenko, 118-1-04 (F)			KJ802048		
50	Gymnocolea inflata (Huds.) Dumort.	*Gymnocolea inflata* (Huds.) Dumort.	Norway, A. Seneca & L. Soederstroem, Soederstroem 2004/223 (BOL)					AM398321
51	*Hamatostrepta* *concinna* Váňa et D.G. Long	*Hamatostrepta* *concinna* Váňa et D.G. Long	Myanmar, Long, D. 34854 (DUKE)				KF852407	
52	*Hattoria yakushimensis* (Horik.) R.M. Schust.	*Hattoria yakushimensis* (Horik.) R.M. Schust.	Japan, Kagoshima, Yakushima Island, T. Katagiri 4281 (NICH)				LC376047	
53	*Hattoria yakushimensis* (Horik.) R.M. Schust.	*Hattoria yakushimensis* (Horik.) R.M. Schust.	China, Xia Tang et Rui-Ping Shi, July 2017, 20170706-94, COMPLETE CHLOROPLAST GENOME				MW429512	
54	*Heterogemma capitata* (Hook.) Konstant. et Vilnet	*Heterogemma capitata* (Hook.) Konstant. et Vilnet	Russia, N. Konstantinova 121-03 (F)					KF851414
55	*Isopaches bicrenatus* (Schmidel ex Hoffm.) H. Buch	*Isopaches bicrenatus* (Schmidel ex Hoffm.) H. Buch	USA, Vermont, B. Shaw 6970 (DUKE)				KF852384	
56	*Isopaches decolorans* (Limpr.) H. Buch	*Isopaches decolorans* (Limpr.) H. Buch	India, D. Long, 1995, Long 22566 (E)					AM398300
57	*Lophozia bicrenata* (Schmidel ex Hoffm.) Dumort.	*Isopaches bicrenatus* (Schmidel ex Hoffm.) H. Buch	Finland, L. Soederstroem & P. Manyanga, Soederstroem 2003/100 (BOL)					AM398355
58	*Lophozia ascendens* (Warnst.) R.M. Schust.	*Lophozia ascendens* (Warnst.) R.M. Schust.	Russia, Buryatiya, N. Konstantinova, 109-3-01 (KPABG)	DQ875089	DQ875054			
59	*Lophozia ascendens* (Warnst.) R.M. Schust.	*Lophozia ascendens* (Warnst.) R.M. Schust.	Sweden, L. Soederstroem & P. Manyanga, Soederstroem 2003/077 (BOL)			AM397796		
60	*Lophozia ascendens* (Warnst.) R.M. Schust.	*Lophozia ascendens* (Warnst.) R.M. Schust.	Poland, Klama et al. 171 (GOET)				KC184727	
61	*Lophozia austrosibirica* Bakalin	*Lophozia austrosibirica* Bakalin	Russia, Buryatiya, V. Bakalin, 15-9-99 (KPABG)	DQ875105	DQ875069			
62	*Lophozia ciliata* Damsh., L. Söderstr. et H. Weibull	*Lophozia ciliata* Damsh., L. Söderstr. et H. Weibull	Sweden, L. Soederstroem & P. Manyanga, Soederstroem 2003/084 (BOL)			AM397797		
63	*Lophozia excisa* (Dicks.) Dumort.	*Lophoziopsis excisa* (Dicks.) Konstant. et Vilnet	Svalbard, Spitsbergen, N.A. Konstantinova, 104-1-04 (KPABG)	DQ875091	DQ875056			
64	*Lophozia excisa* (Dicks.) Dumort.	*Lophoziopsis excisa* (Dicks.) Konstant. et Vilnet	Russia, Maryi-El, N.A. Konstantinova, K437-2-04 (KPABG)	EF065691	EF065684			
65	*Lophozia excisa* (Dicks.) Dumort.	*Lophoziopsis excisa* (Dicks.) Konstant. et Vilnet	Norway, L. Soederstroem & P. Manyanga, Soederstroem 2003/030 (BOL)					AM398371
66	*Lophozia fuscovirens* Bakalin et Vilnet	*Lophozia fuscovirens* Bakalin et Vilnet	Russia, Russian Far East, Kamchatka Territory, V.A. Bakalin, K-52-9-22	**PV822256**	**PV835345**	**PV835351**	**PV835363**	**PV835357**
67	*Lophozia fuscovirens* Bakalin et Vilnet	*Lophozia fuscovirens* Bakalin et Vilnet	Norway, Svalbard, A. Savchenko, CA16-12-1c (KPABG)	MK774737	MK779914			
68	*Lophozia fuscovirens* Bakalin et Vilnet	*Lophozia fuscovirens* Bakalin et Vilnet	Russia, Magadan Prov., V.A. Bakalin, Mag-28-32-13 (KPABG)	MK007091	MK012211			
69	*Lophozia fuscovirens* Bakalin et Vilnet	*Lophozia fuscovirens* Bakalin et Vilnet	Russia, Magadan Prov., V.A. Bakalin, Mag-30-4-14 (KPABG)	MK007093	MK012213			
70	*Lophozia fuscovirens* Bakalin et Vilnet	*Lophozia fuscovirens* Bakalin et Vilnet	Russia, Magadan Prov., V.A. Bakalin, Mag-50-16-11 (KPABG)	MK007092	MK012212			
71	*Lophozia incisa* (Schrad.) Dumort.	*Schistochilopsis incisa* (Schrad.) Konstant.	Sweden, L. Soederstroem & P. Manyanga, Soederstroem 2003/079 (BOL)			AM397800		AM398366
72	*Lophozia koreana* (Bakalin, S.S. Choi et B.Y. Sun) Maltseva, Vilnet et Bakalin	*Lophozia koreana* (Bakalin, S.S. Choi et B.Y. Sun) Maltseva, Vilnet et Bakalin	Republic of Korea, South Korea, Jeollabuk-do, V.A. Bakalin & S.S. Choi, Kor-74-5-19	MW685441	MW654174	MW654177	**PV853827**	**PV853830**
73	*Lophozia koreana* (Bakalin, S.S. Choi et B.Y. Sun) Maltseva, Vilnet et Bakalin	*Lophozia koreana* (Bakalin, S.S. Choi et B.Y. Sun) Maltseva, Vilnet et Bakalin	Republic of Korea, South Korea, Jeollabuk-do, V.A. Bakalin & S.S. Choi, Kor-75-15-19	MW685442	MW654175	MW654178	**PV853828**	**PV853831**
74	*Lophozia koreana* (Bakalin, S.S. Choi et B.Y. Sun) Maltseva, Vilnet et Bakalin	*Lophozia koreana* (Bakalin, S.S. Choi et B.Y. Sun) Maltseva, Vilnet et Bakalin	Republic of Korea, South Korea, Jeollabuk-do, V.A. Bakalin & S.S. Choi, Kor-76-1-19	MW685443	MW654176	MW654179	**PV853829**	**PV853832**
75	*Lophozia lantratovae* Bakalin	*Lophozia lantratovae* Bakalin	Russia, Buryatiya, V.A. Bakalin, 76-7-01 (KPABG)	DQ875090	DQ875055			
76	*Lophozia lantratovae* Bakalin	*Lophozia lantratovae* Bakalin	China, Sichuan Prov., V.A. Bakalin & K.G. Klimova, China-39-3-17 (VBGI)	MK007087				
77	*Lophozia lantratovae* Bakalin	*Lophozia lantratovae* Bakalin	Russia, North Ossetia, Alanya Rep., A.V. Rumyantseva, 123153 (KPABG)	MW872337	MW887533			
78	*Lophozia lantratovae* Bakalin	*Lophozia lantratovae* Bakalin	Russia, Russian Far East, V.A. Bakalin, P-72-19-05				KC184728	
79	*Lophozia longidens* (Lindb.) Macoun	*Lophoziopsis longidens* (Lindb.) Konstant. et Vilnet	Norway, L. Soederstroem, Soederstroem 2003/016 (BOL)					AM398340
80	*Lophozia obscura* (Bakalin) A.V. Troitsky, Bakalin et Fedosov	*Lophozia obscura* (Bakalin) A.V. Troitsky, Bakalin et Fedosov	Russia, Far East, Sakhalin Province, V.A. Bakalin, K-79-21-15 Sc21/0 (VBGI)	MT431697	MT381919	MT381885		
81	*Lophozia obtusa* (Lindb.) A. Evans	*Obtusifolium obtusum* (Lindb.) S.W. Arnell	Finland, Soederstroem 2003/094 (BOL)					AM398359
82	*Lophozia pacifica* Bakalin	*Lophozia pacifica* Bakalin	Russia, Russian Far East, Kamchatka Territory, V.A. Bakalin & K.G. Klimova, K-107-38-21	**PV822257**	**PV835344**			
83	*Lophozia pacifica* Bakalin	*Lophozia pacifica* Bakalin	Russia, Kamchatka Terr., V.A. Bakalin, K-16-2-02-VB (KPABG)	MK007089	MK012209			
84	*Lophozia dubia* Schiffn.	*Lophozia dubia* Schiffn.	China, Yunnan Province, Diqing Prefecture, V.A. Bakalin & W.Z. Ma, C-83-7-18	OR982393	OR995729	**PV835348**	**PV835360**	**PV835354**
85	*Lophozia pellucida* R.M. Schust.	*Lophoziopsis pellucida* (R.M. Schust.) Konstant. et Vilnet	Russia, Murmanskaya Prov., N.A. Konstantinova, 39-2a-03 (KPABG)	EF065695	EF065687			
86	*Lophozia perssonii* H. Buch et S.W. Arnell	*Oleolophozia perssonii* (H. Buch et S.W. Arnell) L. Söderstr., De Roo et Hedd.	Norway, Soederstroem 2003/036 (BOL)					AM398374
87	*Lophozia propagulifera* (Gottsche) Steph.	*Lophoziopsis* *propagulifera* (Gottsche) Konstant. et Vilnet	Russia, Kamchatskaya Prov., V.A. Bakalin, K-53-6-02-VB (KPABG)		DQ875062			
88	*Lophozia setosa* (Mitt.) Steph.	*Schistochilopsis setosa* (Mitt.) Konstant.	Bhutan, D. Long 28644 (E)			AM397752		AM398317
89	*Lophozia silvicola* H. Buch	*Lophozia silvicola* H. Buch	Russia, Sakhalin Prov., Sakhalin Isl., V.A. Bakalin, S-14-4-17 (VBGI)	MK007090	MK012210			
90	*Lophozia silvicoloides* N. Kitag.	*Lophozia silvicoloides* N. Kitag.	Russia, Murmanskaya Prov., N.A. Konstantinova, 356-4-00 (KPABG)	DQ875099	DQ875064			
91	*Lophozia* sp.	*Lophozia neglecta* Bakalin, Maltseva, Klimova, S.S. Choi, W.Z. Ma sp. nov.	China, Yunnan Province, Diqing Prefecture, V.A. Bakalin & W.Z. Ma, C-86-1-18	**PV822252**	**PV835339**	**PV835346**	**PV835358**	**PV835352**
92	*Lophozia* sp.	*Lophozia neglecta* Bakalin, Maltseva, Klimova, S.S. Choi, W.Z. Ma sp. nov.	China, Yunnan Province, Diqing Prefecture, V.A. Bakalin & W.Z. Ma, C-86-2-18	**PV822253**	**PV835340**	**PV835347**	**PV835359**	**PV835353**
93	*Lophozia sudetica* (Nees ex Huebener) Grolle	*Barbilophozia sudetica* (Nees ex Huebener) L. Söderstr., De Roo et Hedd.	Russia, Murmanskaya Prov., V.A. Bakalin, 4.06.1998 (KPABG)					
94	*Lophozia sudetica* (Nees ex Huebener) Grolle	*Barbilophozia sudetica* (Nees ex Huebener) L. Söderstr., De Roo et Hedd.	Russia, Kemerovskaya Prov., N.A. Konstantinova, 90-7-00 (KPABG)	DQ875114				
95	*Lophozia sudetica* (Nees ex Huebener) Grolle	*Barbilophozia sudetica* (Nees ex Huebener) L. Söderstr., De Roo et Hedd.	Norway, L. Soederstroem & P. Manyanga, Soederstroem 2003/049 (BOL)			AM397783		AM398351
96	*Lophozia svalbardensis* Konstant., Vilnet et Mamontov	*Lophozia svalbardensis* Konstant., Vilnet et Mamontov	Russia, Krasnoyarsk Territory, Kara Sea, Izvestii Tsik Islands, Sverdrup Island, 10 September 2021, I.V. Czenyadjeva, 28-21, LE-B0028582	PQ270121	PQ283676			
97	*Lophozia svalbardensis* Konstant., Vilnet et Mamontov	*Lophozia svalbardensis* Konstant., Vilnet et Mamontov	Norway, Svalbard, N.A. Konstantinova, K-135-3a-07 (KPABG)	MW298768	MW297149			
98	*Lophozia ventricosa* (Dicks.) Dumort.	*Lophozia ventricosa* (Dicks.) Dumort.	United Kingdom, Scotland, Long, D.G. 31226 (E)				KF852282	JF513477
99	*Lophozia ventricosa* var. *confertifolia* (Schiffn.) Husn.	*Lophozia wenzelii* (Nees) Steph.	France, Vaňa s.n. (BOL)			AM397734		AM398287
100	*Lophozia ventricosa* var. *confusa* R.M. Schust.	*Lophozia ventricosa* (Dicks.) Dumort.	Canada, Hedderson 5008 (BOL)			AM397801		AM398368
101	*Lophozia ventricosa* var. *longiflora* (Nees) Macoun	*Lophozia longiflora* (Nees) Schiffn.	Russia, Chitinskaya Prov., V.A. Bakalin, 11-5-00 (KPABG)	DQ875101				
102	*Lophozia wenzelii* var. *groenlandica* (Nees) Bakalin	*Lophozia wenzelii* (Nees) Steph.	Russia, Murmanskaya Prov., N.A. Konstantinova, 9329 (KPABG)	DQ875109	DQ875073			
103	*Lophozia wenzelii* var. *lapponica* H. Buch et S.W. Arnell	*Lophozia wenzelii* var. *lapponica* H. Buch et S.W. Arnell	Svalbard, Spitsbergen, N. Konstantinova, 124-2-04	DQ875112	DQ875076			
104	*Lophozia wenzelii* var. *massularioides* Bakalin	*Lophozia wenzelii* var. *massularioides* Bakalin	Russia, Caucasus, Onipchenko, 31.08.83 (MHA)	DQ875111	DQ875075			
105	*Lophoziopsis excisa* (Dicks.) Konstant. et Vilnet	*Lophoziopsis excisa* (Dicks.) Konstant. et Vilnet	United Kingdom, Scotland, Long, D.G. 35611 (E)				KF852273	KF851381
106	*Lophoziopsis excisa* (Dicks.) Konstant. et Vilnet	*Lophoziopsis excisa* (Dicks.) Konstant. et Vilnet	Russia, Russian Far East, V.A. Bakalin, P-74-17-05				KC184729	
107	*Lophoziopsis longidens* (Lindb.) Konstant. et Vilnet	*Lophoziopsis longidens* (Lindb.) Konstant. et Vilnet	Russia, Murmanskaya Prov., N.A. Konstantinova, 360-2-00 (KPABG)	DQ875094	DQ875059			
108	*Lophoziopsis pellucida* (R.M. Schust.) Konstant. et Vilnet	*Lophoziopsis pellucida* (R.M. Schust.) Konstant. et Vilnet	Russia, Trans-Baikal Terr., Yu.S. Mamontov, 356-3-6 (KPABG)	MW298773	MW297154			
109	*Lophoziopsis polaris* (R.M. Schust.) Konstant. et Vilnet	*Lophoziopsis polaris* (R.M. Schust.) Konstant. et Vilnet	Norway, Svalbard, N.A. Konstantinova, A.N. Savchenko, K129-07 (KPABG)	MT334459	MT338482			
110	*Lophoziopsis polaris* var. *sphagnorum* (R.M. Schust.) Konstant. et Vilnet	*Lophoziopsis polaris* var. *sphagnorum* (R.M. Schust.) Konstant. et Vilnet	Russia, Yakutiya, V.A. Bakalin, 23-11-00 (KPABG)	DQ875096				
111	*Neoorthocaulis* *attenuatus* (Mart.) L. Söderstr., De Roo et Hedd.	*Neoorthocaulis* *attenuatus* (Mart.) L. Söderstr., De Roo et Hedd.	Russia, Kamchatka Terr., V.A. Bakalin, 66-1-01-VB (KPABG)	OR769958	OR762252			
112	*Neoorthocaulis* *attenuatus* (Mart.) L. Söderstr., De Roo et Hedd.	*Neoorthocaulis* *attenuatus* (Mart.) L. Söderstr., De Roo et Hedd.	Poland, Strebel 226 (GOET)				KC184733	
113	*Neoorthocaulis floerkei* (F. Weber et D. Mohr) L. Söderstr., De Roo et Hedd.	*Neoorthocaulis floerkei* (F. Weber et D. Mohr) L. Söderstr., De Roo et Hedd.	Canada, British Columbia, B Shaw F699 (DUKE)					KF851449
114	*Neoorthocaulis floerkei* (F. Weber et D. Mohr) L. Söderstr., De Roo et Hedd.	*Neoorthocaulis floerkei* (F. Weber et D. Mohr) L. Söderstr., De Roo et Hedd.	United Kingdom, Scotland, Berwickshire, near Raecleugh, Long 31215 (E)				KC297118	
115	*Neoorthocaulis floerkei* (F. Weber et D. Mohr) L. Söderstr., De Roo et Hedd.	*Neoorthocaulis floerkei* (F. Weber et D. Mohr) L. Söderstr., De Roo et Hedd.	Drehwald s.n. (GOET)					
116	*Oleolophozia perssonii* (H. Buch et S.W. Arnell) L. Söderstr., De Roo et Hedd.	*Oleolophozia perssonii* (H. Buch et S.W. Arnell) L. Söderstr., De Roo et Hedd.	Russia, Russian Far East, Magadan Province, Kolyma Upland, V.A. Bakalin, Mag-31-13-11 (VBGI)	OR763333	MT476335	MT482533	OR785042	
117	*Plicanthus birmensis* (Steph. ex Schiffn.) R.M. Schust.	*Plicanthus birmensis* (Steph. ex Schiffn.) R.M. Schust.	Japan, Hiroshima, Ryuzu gorge, K. Amamoto 13 (HIRO)				LC648969	
118	*Plicanthus hirtellus* (F. Weber) R.M. Schust.	*Plicanthus hirtellus* (F. Weber) R.M. Schust.	China, Yunnan, Long, D.G. 34407 (E)				KF852278	
119	*Plicanthus hirtellus* (F. Weber) R.M. Schust.	*Plicanthus hirtellus* (F. Weber) R.M. Schust.	Gradstein 10388 (GOET)				KC184743	
120	Plicanthus hirtellus (F. Weber) R.M. Schust.	Plicanthus hirtellus (F. Weber) R.M. Schust.	Japan, Gifu, Mt. Kinka, K. Amamoto 10 (HIRO)				LC648968	
121	*Plicanthus hirtellus* (F. Weber) R.M. Schust.	*Plicanthus hirtellus* (F. Weber) R.M. Schust.	Nepal, D. Long, Long 30335 (E)					AM398304
122	*Pseudolophozia debiliformis* (R.M. Schust. et Damsh.) Konstant. et Vilnet	*Barbilophozia sudetica* (Nees ex Huebener) L. Söderstr., De Roo et Hedd.	Svalbard, Spitsbergen, N.A. Konstantinova, K 91-3-06 (KPABG)	HQ897045				
123	*Pseudolophozia* sp.	*Barbilophozia* sp.	Russia, Russian Far East, Sakhalin Province, V.A. Bakalin & K.G. Klimova, K-34-25-18		MT476339	MT482525		MT482518
124	*Pseudolophozia sudetica* (Nees ex Huebener) Konstant. et Vilnet	*Barbilophozia sudetica* (Nees ex Huebener) L. Söderstr., De Roo et Hedd.	Russia, Caucasus, N.A. Konstantinova, K 334/10-08 (KPABG)	HQ897042	HQ897153	HQ897090		
125	*Pseudolophozia sudetica* (Nees ex Huebener) Konstant. et Vilnet	*Barbilophozia sudetica* (Nees ex Huebener) L. Söderstr., De Roo et Hedd.	Russia, Komi Rep., M.V. Dulin, 110215 (KPABG)	HQ897001	HQ897112	HQ897089		
126	*Pseudolophozia sudetica* (Nees ex Huebener) Konstant. et Vilnet	*Barbilophozia sudetica* (Nees ex Huebener) L. Söderstr., De Roo et Hedd.	Russia, Perm Territory, N.A. Konstantinova, 108221 (KPABG)	HQ897002				
127	*Scapania aequiloba* (Schwägr.) Dumort.	*Scapania aequiloba* (Schwägr.) Dumort.	Czech Republic, B. Shaw 12990 (DUKE)				KC297122	
128	*Scapania cuspiduligera* (Nees) Müll. Frib.	*Scapania cuspiduligera* (Nees) Müll. Frib.	USA, Long 13996 (GOET)				KC184748	
129	*Scapania hyperborea* Jørg.	*Scapania hyperborea* Jørg.	Norway, Hentschel Bryo 03230 (GOET)				KC184749	
130	*Scapania magadanica* S.S. Choi, Bakalin et B.Y. Sun	*Scapania magadanica* S.S. Choi, Bakalin et B.Y. Sun	Russia, Russian Far East, Magadan Province, V.A. Bakalin, Mag-61-40-11 (VBGI)	OP654523	OP641874	OP641886		
131	*Scapania nemorosa* (L.) Dumort.	*Scapania nemorea* (L.) Grolle	USA, Davis 124 (DUKE)		AY608143	AY608190	AY608039	AY608108
132	*Scapania nimbosa* Taylor ex Lehm.	*Scapania nimbosa* Taylor ex Lehm.	United Kingdom, Scotland, Long, D.G & Flagmeier, M. 37028 (DUKE)				KF852408	KF851502
133	*Scapania paludosa* (Müll. Frib.) Müll. Frib.	*Scapania paludosa* (Müll. Frib.) Müll. Frib.	Germany, Schaefer-Verwimp 19613 (GOET)				KC184753	
134	*Scapania stephanii* Müll. Frib.	*Scapania stephanii* Müll. Frib.	Japan, Honshu, Hiroshima, H. Matsuda 26 (HIRO)				AB476597	
135	*Scapania undulata* (L.) Dumort.	*Scapania undulata* (L.) Dumort.	Finland, Nuuksio National Park, 2000 He-Nygren & Piippo 1468		AY149859		AY149840	AY462392
136	*Schistochilopsis incisa* (Schrad.) Konstant.	*Schistochilopsis incisa* (Schrad.) Konstant.	USA, California, Shevock 27788 (GOET)				KC184760	
137	*Schistochilopsis setosa* (Mitt.) Konstant.	*Schistochilopsis setosa* (Mitt.) Konstant.	China, Yunnan, Long, D.G. 34712 (E)				KF852274	
138	*Schljakovia kunzeana* (Huebener) Konstant. et Vilnet	*Schljakovia kunzeana* (Huebener) Konstant. et Vilnet	Russia, Chita Prov., V.A. Bakalin, 38-1-00 (KPABG)		OR762255			
139	*Schljakovianthus quadrilobus* (Lindb.) Konstant. et Vilnet	*Schljakovianthus quadrilobus* (Lindb.) Konstant. et Vilnet	Russia, Murmansk Prov., Yu. Mamontov, YuSM-412-3-1, 400334 (INEP)		OR762256			
140	*Schljakovianthus quadrilobus* (Lindb.) Konstant. et Vilnet	*Schljakovianthus quadrilobus* (Lindb.) Konstant. et Vilnet	USA, Alaska, B Shaw F982b/8 DUKE				KF852393	KF851482
141	*Sphenolobus minutus* (Schreb.) Berggr.	*Sphenolobus minutus* (Schreb.) Berggr.	Norway, Spitsbergen, Hentschel Bryo421 (GOET)	GQ900013			DQ312475	
142	*Sphenolobus saxicola* (Schrad.) Steph.	*Sphenolobus saxicola* (Schrad.) Steph.	Czech Republic, N. Bohemia, B. Buryova 26.9.1995 (DUKE)				KF852357	
143	*Sphenolobus saxicola* (Schrad.) Steph.	*Sphenolobus saxicola* (Schrad.) Steph.	Germany, Heinrichs et al. JH3734 (GOET)				KC184761	
144	*Tetralophozia cavallii* (Gola) Váňa	*Tetralophozia cavallii* (Gola) Váňa	Tanzania, Pocs & Ochyra 88152/C (GOET)				KC184762	
145	*Tetralophozia filiformis* (Steph.) Urmi	*Tetralophozia filiformis* (Steph.) Urmi	China, Yunnan, B. Shaw 5790 (DUKE)				KF852352	
146	*Tetralophozia filiformis* (Steph.) Urmi	*Tetralophozia filiformis* (Steph.) Urmi	China, Sichuan Prov., Bakalin & Klimova, China-39-1-17 (VBGI)	MZ231275	MZ229433			
147	*Tetralophozia pilifera* (Steph.) R.M. Schust.	*Tetralophozia pilifera* (Steph.) R.M. Schust.	Indonesia, Gradstein 11015 (GOET)				KC184763	
148	*Tetralophozia pusilla* (Steph.) Bakalin et Vilnet	*Tetralophozia pusilla* (Steph.) Bakalin et Vilnet	Japan, Yamanashi Pref., V.A. Bakalin, J-88-40-15 (VBGI) (Tl1+2/12+13)	MZ231278	MZ229436	MZ229442		
149	*Tetralophozia setiformis* (Ehrh.) Schljakov	*Tetralophozia setiformis* (Ehrh.) Schljakov	Canada, Faubert 268.3 (GOET)				KC184764	
150	*Tetralophozia setiformis* (Ehrh.) Schljakov	*Tetralophozia setiformis* (Ehrh.) Schljakov	Sweden, L. Soederstroem & P. Manyanga, Soederstroem 2003/056 (BOL)					AM398370
151	*Tetralophozia setiformis* (Ehrh.) Schljakov	*Tetralophozia setiformis* (Ehrh.) Schljakov	Norway, Soederstroem 2004/195 (BOL)					AM398277
152	*Trilophozia quinquedentata* (Huds.) Bakalin	*Trilophozia quinquedentata* (Huds.) Bakalin	Norway, Svalbard, N.A. Konstantinova & A.N. Savchenko, K70-08 (KPABG)	MT334460				
153	*Tritomaria exsecta* (Schmidel) Schiffn. ex Loeske	*Tritomaria exsecta* (Schmidel) Schiffn. ex Loeske	Russia, Nijegorodskaya Prov., N.A. Konstantinova, 103-1-03 (KPABG)	EU791800	EU791682			
154	*Tritomaria exsecta* (Schmidel) Schiffn. ex Loeske	*Tritomaria exsecta* (Schmidel) Schiffn. ex Loeske	Russia, Siberia, Schaefer-Verwimp & Verwimp 21816 (GOET)				KC184765	
155	*Tritomaria* *exsectiformis* (Breidl.) Schiffn. ex Loeske	*Lophozia vinacea* Bakalin, Maltseva, Klimova, S.S. Choi, W.Z. Ma sp. nov.	China, Yunnan Province, Diqing Prefecture, V.A. Bakalin & W.Z. Ma, C-83-24a-18	**PV822254**	**PV835342**	**PV835349**	**PV835361**	**PV835355**
156	*Tritomaria* *exsectiformis* (Breidl.) Schiffn. ex Loeske	*Lophozia vinacea* Bakalin, Maltseva, Klimova, S.S. Choi, W.Z. Ma sp. nov.	China, Yunnan Province, Diqing Prefecture, V.A. Bakalin & W.Z. Ma, C-83-26-18	**PV822255**	**PV835343**	**PV835350**	**PV835362**	**PV835356**
157	*Tritomaria* *exsectiformis* (Breidl.) Schiffn. ex Loeske	*Tritomaria* *exsectiformis* (Breidl.) Schiffn. ex Loeske	Russia, Rep. Buryatiya, N.A. Konstantinova, 83-4-01 (KPABG)		EU791683			
158	*Tritomaria* *exsectiformis* (Breidl.) Schiffn. ex Loeske	*Tritomaria* *exsectiformis* (Breidl.) Schiffn. ex Loeske	Russia, Siberia, Schaefer-Verwimp & Verwimp 21849/A (GOET)				KC184766	
159	*Tritomaria ferruginea* (Grolle) Váňa	*Tritomaria ferruginea* (Grolle) Váňa	China, Yunnan, B. Shaw 5763 (DUKE)					KF851443
160	*Tritomaria polita* (Nees) Jørg.	*Saccobasis polita* (Nees) H. Buch	Norway, L. Soederstroem & P. Manyanga, Soederstroem 2003/037 (BOL)					AM398342
161	*Tritomaria polita* (Nees) Jørg.	*Saccobasis polita* (Nees) H. Buch	Sweden, L. Soederstroem, Soederstroem 2003/063 (BOL)					AM398376
162	*Tritomaria quinquedentata* (Huds.) H. Buch	*Trilophozia quinquedentata* (Huds.) Bakalin	Poland, 41204 (POZW)			JQ658789		
163	*Tritomaria quinquedentata* (Huds.) H. Buch	*Trilophozia quinquedentata* (Huds.) Bakalin	Russia, N.A. Konstantinova & A.N. Savchenko 515-3-05 (F)				KF852302	KF851406
164	*Tritomaria quinquedentata* (Huds.) H. Buch	*Trilophozia quinquedentata* (Huds.) Bakalin	Finland, Lammi, 2003 He-Nygren & Piippo 1474		AY463592		AY462334	AY462400
165	*Tritomaria quinquedentata* f. *gracilis* R.M. Schust.	*Trilophozia quinquedentata* f. *gracilis* (R.M. Schust.) Konstant.	Svalbard, North-East Land, N.A. Konstantinova, K 72-2-06 (KPABG)	EU791803	EU791685			
166	*Tritomaria scitula* (Taylor) Jørg.	*Tritomaria scitula* (Taylor) Jørg.	Russia, Komi Rep., M. Dulin, N.A. Konstantinova, G101301 (KPABG)	EU791799				
167	*Tritomaria scitula* (Taylor) Jørg.	*Tritomaria scitula* (Taylor) Jørg.	Norway, L. Soederstroem & P. Manyanga, Soederstroem 2003/028 (BOL)					AM398347
168	*Vietnamiella* *epiphytica* Bakalin et Vilnet	*Vietnamiella* *epiphytica* Bakalin et Vilnet	Vietnam, Lao Cai Prov., V.A. Bakalin & K.G. Klimova, V-9-7-17 (VBGI)	MK277316	MK290984		MK290986	
169	*Vietnamiella epiphytica* Bakalin et Vilnet	*Vietnamiella epiphytica* Bakalin et Vilnet	Vietnam, Lao Cai Prov., V.A. Bakalin & K.G. Klimova, V-9-8-17 (VBGI)	MK277317	MK290985			
